# Diversity in Psychological Research Activities: Quantitative Approach With Topic Modeling

**DOI:** 10.3389/fpsyg.2021.773916

**Published:** 2021-12-16

**Authors:** Sachio Otsuka, Yoshiyuki Ueda, Jun Saiki

**Affiliations:** ^1^Graduate School of Human and Environmental Studies, Kyoto University, Kyoto, Japan; ^2^Kokoro Research Center, Kyoto University, Kyoto, Japan

**Keywords:** diversity, regional difference, publication period, text mining, topic modeling

## Abstract

Recent cultural studies have discussed universality and diversity in human behavior using numerous samples investigated worldwide. We aimed to quantitatively extend this discussion to various research activities in psychology in terms of geographic regions and time trends. Most psychology departments have specialists in various fields of psychology. Further, research institutions in all regions typically aim to provide systematic and balanced research education. Nevertheless, most researchers recognize universal features and patterns of diversity in research activities in psychology in terms of regional differences and time trends. However, these arguments remain intuitive and vague, and no studies have conducted quantitative analyses. To this end, we conducted topic modeling for the abstracts of psychological articles with the regions of author affiliations and publication periods as covariates. The results showed that the topic proportions related to basic research were high in North-Central America, whereas those related to clinical research were high in Europe. Interestingly, the regional differences shown by topic modeling were not observed in the frequency analysis of keywords, indicating that topic modeling revealed implicit characteristics. Moreover, we observed an increasing trend of neuroscience topics across publication periods. However, this trend was not valid for the psychology journal *Psychological Science*. Taken together, our results suggest diversity of geographic regions and periods in research activities in psychology. More importantly, our findings indicate that universality holds neither for human behavior nor research activities on human mental processes.

## Introduction

It is becoming increasingly important to recognize diversity in various areas, such as race issues, political activities, and sciences (Mallapaty, 2019; Forrester, 2020). In psychology, for over 10 years, it has been discussed that the findings from psychological research are primarily based on samples drawn from Western, educated, industrialized, rich, and democratic (WEIRD) societies and that research activities are concentrated in the United States of America (Henrich et al., 2010). Previous studies have demonstrated that people in Western and East Asian cultures are affected by cultural differences not only in higher-order cognition, such as self-concept and moral judgment (Markus and Kitayama, 1991; Awad et al., 2020), but also in fundamental visual perception (Kitayama et al., 2003; Ueda et al., 2018). In contrast, emotional expression is universal across populations (Ekman, 1999; Tracy and Matsumoto, 2008: but see Barrett, 2017). Therefore, researchers must be aware of whether the findings resulting from their studies can be generalized to *Homo sapiens* worldwide (Henrich et al., 2010, 2020; Kupferschmidt, 2019). Recent cultural studies grasped both universality and diversity in human behavior with large-scale investigations worldwide (Mehr et al., 2019; Awad et al., 2020).

In this study, we aimed to extend diversity of geographic regions and time trends to research activities in psychology. Most psychology departments worldwide have specialists in perceptual, cognitive, social, personality, educational, applied, clinical, and developmental psychology, and some psychologists study neuroscientific, computational, and statistical approaches. Further, research institutions in every region aim to provide systematic and balanced psychology research education. Nevertheless, most researchers are probably aware that there are distinctive characteristics in psychological research activities in each region. It may be generally realized by psychologists that North America is sophisticated in basic research in experimental psychology and Europe in applied and clinical research. Moreover, basic research in psychology may be relatively constant across time, and the number of neuroscience studies may consistently increase owing to advancements in measurements and the increasing number of publication venues (e.g., open access journals). However, no studies have quantitatively examined the tacit knowledge described above. To facilitate discussion about appropriate research and education activities in psychology worldwide, quantitative evidence should be provided based on an objective data analysis of whether there is diversity in research activities in psychology at a higher level compared with cultural differences in human behavior and the WEIRD problem.

To this end, we conducted topic modeling, a text mining approach based on probabilistic modeling. Topic modeling with latent Dirichlet allocation (LDA; Blei et al., 2003) is used to identify topics that have a meaningful structure within collections of documents. Text mining approaches with topic modeling have been applied not only to open-ended survey responses (e.g., Roberts et al., 2014; He et al., 2020) but also to abstracts of scientific articles (Griffiths and Steyvers, 2004; Wang et al., 2016; Rubin et al., 2017; Lin et al., 2020). For example, Griffiths and Steyvers (2004) conducted topic modeling with the abstracts of articles published in *Proceedings of the National Academy of Sciences* from 1991 to 2001 and showed that the topics extracted with topic modeling were consistent with the article classifications that the authors had selected. Recently, Wang et al. (2016) used topic modeling for the abstracts of articles related to adolescent substance use and depression and suggested that text mining can be used as a tool to both recapture already known facts and uncover hot and cold topics. More importantly, topic modeling can handle a large-scale dataset of research abstracts from various regions and periods. We conducted two substudies, henceforth referred to as Study 1 and Study 2. In Study 1, we conducted topic modeling using the abstracts of articles related to the Stroop test (Stroop, 1935) and to visual search to examine the article topics. We selected such articles because they concern fundamental tasks that reflect human attentional and cognitive control (e.g., Bugg, 2012; Theeuwes, 2019). Furthermore, these techniques are introduced in most psychology textbooks (e.g., Nolen-Hoeksema et al., 2014), and are frequently used in psychological tests in applied, clinical, aging, and neuroscience studies worldwide. In fact, some previous studies have used these psychological tasks in basic research (Schmidt and Besner, 2008; Anderson et al., 2011; Kinoshita et al., 2017; Stilwell et al., 2019), clinical research (Fuller et al., 2006; Harrington et al., 2013; Romero et al., 2015; Michael et al., 2020), developmental and aging research (Madden et al., 2004; Joseph et al., 2009; Linzarini et al., 2015; Delalande et al., 2020), and neuroscience studies (Peelen and Kastner, 2011; Foster et al., 2017; Popov et al., 2018; Ramm et al., 2020). Therefore, we can predict the topics consisting of the words associated with various research activities by applying the articles related to the Stroop test and visual search.

Moreover, we examined whether specific proportions of topics related to these psychological themes differ across geographic regions worldwide. Roberts et al. (2014) introduced structural topic modeling (STM), which can be used by researchers to examine whether the prevalence of a topic is influenced by the covariates attached to each document (e.g., author’s gender). In Study 2, we conducted STM using the abstracts examined in Study 1 as documents and taking the geographic region of the first author’s affiliation as a covariate of the topic occurrence. Additionally, we examined if time trends were present in psychological themes. To this end, we conducted another STM using the publication year of each research as a covariate of the topic occurrence. Furthermore, we examined whether the regional differences in the research activities observed in STM of the abstracts are observed in the frequencies of keywords of each manuscript. Finally, we conducted an additional STM to examine whether the trends of research activities found using STM are observed in a given journal (namely, *Psychological Science*).

This study is the first quantitative test of diversity of geographic regions and time trends in research activities in psychology. We believe that a text mining approach with topic modeling for research abstracts is advantageous in clarifying past and present trends of research topics in different geographic regions and in providing important insights to aid future research activities in psychology.

## Materials and Methods

### Dataset

#### Study 1

We used the keywords “Stroop” and “visual search” to search manuscript titles and abstracts in PubMed on September 28 and October 12, 2020. The search word “Stroop” yielded 7,508, and the phrase “visual search” yielded 5,408 abstracts. The search was conducted using the R packages *rvest* and *tidyverse* (Wickham, 2019, 2020) with R and RStudio software (R Core Team, 2020; RStudio Team, 2020). We removed Editorials, Errata, Author Corrections, and Publisher Corrections from the obtained abstracts. In addition, we removed duplicate abstracts, that is, same abstracts obtained more than once. We also obtained the author names, title, affiliations, keywords, and publication year for Study 2. Missing information was collected from Scopus and each publisher to the maximum extent possible. We used each abstract as text data in the following modeling.

Before preprocessing the abstracts, we removed the irrelevant descriptions related to the copyright and trial registration (e.g., “PsycInfo Database Record (c) 2020 APA, all rights reserved”). To preprocess the text data, we used the R package *tm* (Feinerer et al., 2008). All capital letters were transformed into lowercase letters. We removed all punctuation marks, numerical digits, symbols, such as “_” and “.,” and white spaces. Moreover, we changed “colour” to “color” in all abstracts. Then, we used the *stemDocument* function to stem words in the text document. We removed stop words, such as “a” and “the” using the R package *stopwords* (Benoit et al., 2020). In addition, general words used in scientific abstracts, such as “background,” “aim,” “introduction,” “method,” “result,” and “conclusion,” were removed from the dataset. “Stroop,” “visual,” and “search” were also removed from the dataset to avoid poor discriminative information because these words should be retrieved from almost all abstracts. After the above preprocessing of the text data, we created document-term-matrix data for the subsequent topic modeling (i.e., bag of words). We removed the words whose frequencies were less than five by using the R package *gofastr* (Rinker, 2017). Finally, we used 7,028 words for the Stroop test and 4,766 words for visual search for topic modeling.

#### Study 2

We used the abstracts of the papers related to the Stroop test and visual search utilized in Study 1. In addition, we used the data of geographic regions of the first author’s affiliation as the metadata of STM. Supplementary Table 1 shows the number of abstracts related to the Stroop test in each country of the first affiliation of the first author. We classified the countries of the first affiliation of the first author into the following six regions: North-Central America, South America, Europe, Africa, Asia, and Oceania. The numbers of abstracts related to the Stroop test in each region were as follows: North-Central America: 2,551; South America: 201; Europe: 3,073; Africa: 37; Asia: 1,389; and Oceania: 257. Moreover, the numbers of abstracts related to visual search in each region were as follows: North-Central America: 2,245; South America: 30; Europe: 2,274; Africa: 2; Asia: 632; and Oceania: 225 (Supplementary Table 2). Some previous studies on STM have stated that the sample size corresponding to the covariates (i.e., metadata) should be as consistent as possible (Hu et al., 2019; He et al., 2020). Because of the small size of South America, Africa, and Oceania, we selected the datasets of North-Central America, Europe, and Asia in the following STM. In other words, we used 7,013 abstracts of the Stroop test and 5,151 abstracts of visual search. We conducted the same preprocessing for the abstracts as in Study 1. We used 6,768 words for the Stroop test and 4,642 words for visual search for STM with three geographic regions as metadata.

Furthermore, we used the publication year of each manuscript as additional metadata of the STM. Supplementary Figure 1 shows the number of manuscripts related to the Stroop test and visual search published in each year (see also Supplementary Tables 3 and 4). Because of the small number of publications in the early period, we created publication periods so that each period contained at least 200 manuscripts. In the Stroop test, we defined six publication periods before 2005: before 1990 was defined as 1989 (225 abstracts), 1990–1994 as 1994 (227 abstracts), 1995–1997 as 1997 (218 abstracts), 1998–2000 as 2000 (296 abstracts), 2001–2002 as 2002 (266 abstracts), and 2003–2004 as 2004 (316 abstracts). From 2005, we used each publication year. In visual search, we defined nine publication periods before 2010: before 1986 was defined as 1985 (213 abstracts), 1986–1992 as 1992 (203 abstracts), 1993–1996 as 1996 (208 abstracts), 1997–1999 as 1999 (203 abstracts), 2000–2001 as 2001 (216 abstracts), 2002–2003 as 2003 (205 abstracts), 2004–2005 as 2005 (290 abstracts), 2006–2007 as 2007 (312 abstracts), and 2008–2009 as 2009 (360 abstracts). From 2010, we used each publication year. Note that we used the same numbers of abstracts and corpora as those used in Study 1 for STM with publication periods as metadata. Again, we conducted the same preprocessing for the abstracts as in Study 1.

### Topic Modeling

#### Study 1

**Figure 1:**
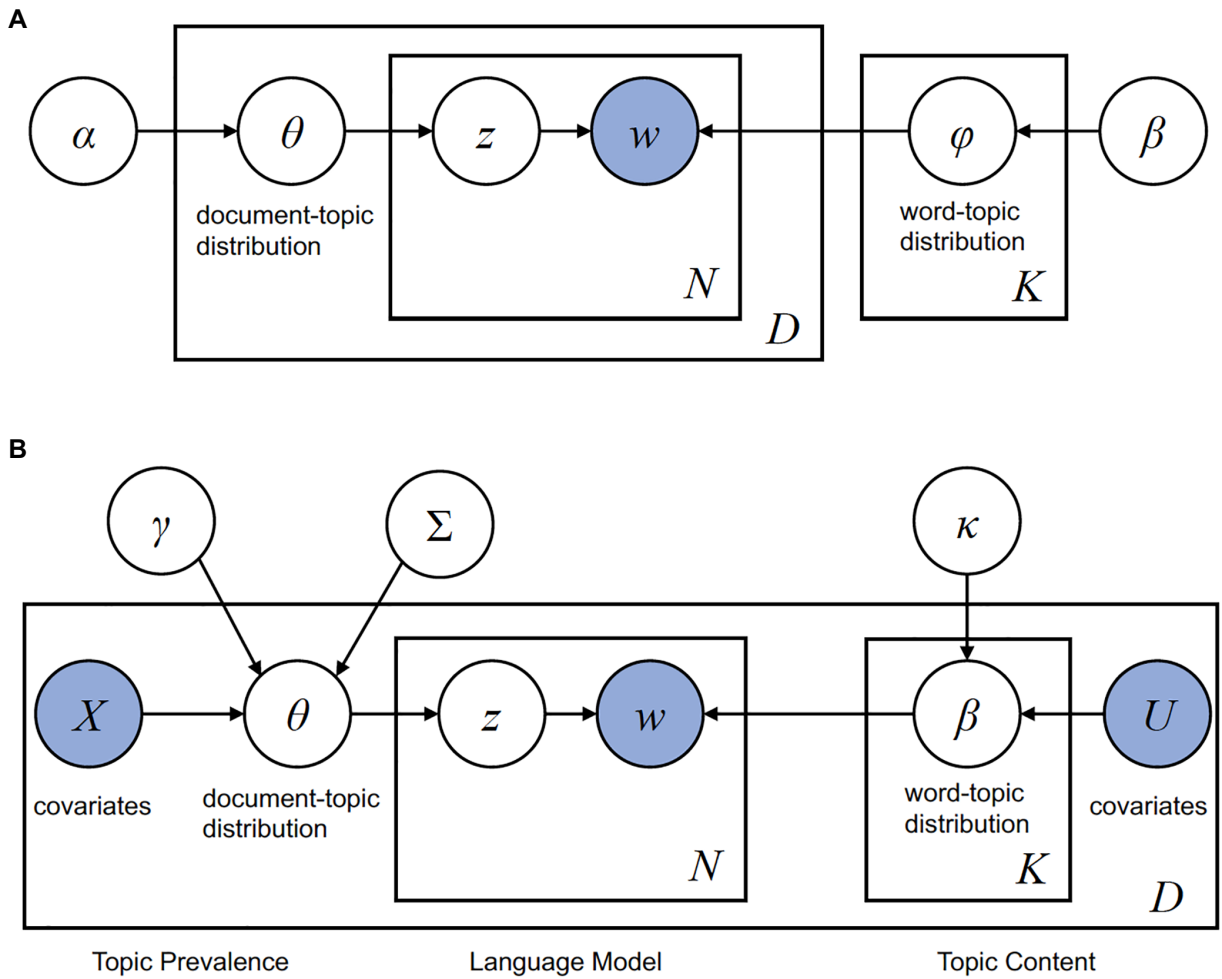
Graphical models of topic modeling. **(A)** A graphical model of topic modeling with latent Dirichlet allocation (LDA; Blei et al., 2003). The rectangles represent repetition; the right square indicates that there is a word distribution (*φ*) corresponding to the number of topics *K* decided by users; the outer left square indicates the number of documents (*D*); the inner left square indicates that there is a number of words (*N*) in each document. The word distribution of the topic is *φ*, which is subject to the parameter *β* of the Dirichlet distribution. The topic distribution of the document is *θ*, which is subject to the parameter *α* of the Dirichlet distribution. The shaded node and unshaded nodes represent the observed and hidden variables, respectively. In other words, only the word *w* in the document can be observed. **(B)** A graphical model of structural topic modeling (STM; Roberts et al., 2014). The flow of STM is the same as that of LDA topic modeling, except that the STM has prior structure information on generalized linear models with covariates of each document, *Χ* and *U*, as parameters (*cf.* Roberts et al., 2014; He et al., 2020): *Χ_d_* is a 1-by-*p* vector, *γ* is a *p*-by-*K* - 1 matrix of coefficients, and Σ is *K* - 1-by- *K* - 1 covariate matrix. The shaded nodes and unshaded nodes represent the observed and hidden variables, respectively. In STM, the word *w* in the document and covariates of topic prevalence and topic content can be observed.

Topic modeling is used to identify the latent topics in a document and to overview the topics in collections of documents. We performed LDA, which is a popular topic modeling and unsupervised machine learning method, to determine the underlying semantic structure of the documents including text data (Blei et al., 2003). LDA assumes that a set of documents consists of multiple topics and that each document contains at least one topic. A topic is defined as a distribution of frequencies over a fixed vocabulary (*φ_k_*), and each word in each document (*w_dn_*) is drawn from one of the vocabularies of the topics that appear in the document. The presence of topics follows the *θ_d_* distribution. Figure 1A shows a graphical model of LDA topic modeling. LDA assumes the following two-step generative process.

Step 1. For topic *k* = 1, …, *K*

- Generate a distribution over words *φ_k_* ~ Dirichlet (*β*)

Step 2. For document *d* = 1, …, *D*

(a) Generate a distribution over topics *θ_d_* ~ Dirichlet (*α*)
(b) For word *n* = 1, …, *N*
   (i) Generate a topic *z_dn_* ~ Multinominal (*θ_d_*)
   (ii) Generate a word *w_dn_* ~ Multinominal (*φz_dn_*)

LDA was performed using the R package *topicmodels* (Grün and Hornik, 2011). It is critical to choose the appropriate number of topics, and a recent study suggested a way to decide the number of topics by using the rate of perplexity change (Zhao et al., 2015). In this study, we used the R package *ldatuning* (Nikita, 2020) to determine the optimal number of topics before conducting topic modeling. Models on the following number of topics were tested: 10, 20, 30, 40, 50, 60, 70, 80, 90, 100, 120, 140, 160, 180, 200, 250, 300, and 350 topics. According to smaller values on indices from Arun et al. (2010) and Cao et al. (2009), and larger values on indices from Deveaud et al. (2014) and Griffiths and Steyvers (2004), we decided that 80 and 70 topics were optimal for the models of the Stroop test and visual search, respectively (Supplementary Figure 2). However, previous studies tested topic models with multiple numbers of topics (Wang et al., 2016; Lin et al., 2020: e.g., 5, 20, and 50 topics) and examined the words in each topic from different models to choose the optimal number of topics. Considering these studies, we tested topic models with 5, 20, 50 topics and the number of topics suggested by the R package *ldatuning* and compared the contents of words in each topic among the different models. We conducted LDA topic modeling with the following parameters: method = Gibbs (the method to be used for fitting is Gibbs sampling); iteration = 5,000; burn-in = 2,000; thin = 100; seed = 123 (to replicate the results of machine learning); the other parameters were on the default setting of *topicmodels*.

#### Study 2

We conducted STM (Roberts et al., 2014) to examine whether the proportions of topics on the abstracts of the Stroop test and visual search differed among the geographic regions and publication periods. The flow of STM is the same as that of LDA topic modeling. The difference from LDA, which shares prior Dirichlet parameters, *α* and *β*, is that STM contains prior structure information on generalized linear models with covariates of each document, *Χ* and *U*, as parameters (*cf.* Roberts et al., 2014; He et al., 2020). In other words, the parameters of STM are random variables drawn from a logistic-normal generalized linear model based on document-level metadata (Figure 1B). LDA topic modeling assumes that documents in the corpus share the same distribution over per-document topic proportions. Therefore, it is difficult to examine whether the topic proportions resulting from LDA differ among the geographic regions and publication periods using regression analysis (Kuhn, 2018; Hu et al., 2019).

We performed STM using the R package *stm* (Roberts et al., 2019) with R software. We used the function *readCorpus* to convert the preprocessed document-term-matrix format to *stm* format. To select the appropriate number of topics, we used the function *searchK*, which is a data-driven approach to select the appropriate number of topics. Models with the following numbers of topics were tested: 10, 20, 30, 40, 50, 60, 70, 80, 90, 100, 120, 140, 160, 180, 200, 250, 300, and 350 topics (Supplementary Figures 3, 4). Considering the results of semantic coherence, residuals, and the estimation results of Study 1, we decided that 80 and 70 topics were optimal for the models of the Stroop test and visual search, respectively. In Study 2, we tested STMs only with 20 topics and the number of topics suggested by the function *searchK* and compared the contents of words in each topic from the two models. Then, we conducted two STMs with the following parameters: prevalence = geographic regions of the first author’s affiliation and publication periods, respectively; seed = 123 (to replicate the results of machine learning); the other parameters were on the default setting of *stm*. Then, to examine the effects of each metadata type on the topic proportions, we estimated a regression using the function *estimateEffect*, where documents were the units, the outcomes were the proportions of each document about topics in STM, and the geographic regions and publication periods were metadata.

### Apparatus

We ran the R-program for topic modeling on a Windows computer (ThinkStation P920, Lenovo), with an Intel^®^ Xeon^®^ Gold 5,118 CPU (2.30 GHz) and 128 GB of RAM.

## Results

### Study 1

We conducted topic modeling for the manuscript abstracts related to the Stroop test and visual search with four topic numbers (5, 20, 50 topics, and the number of topics suggested by the R package *ldatuning*). LDA for the Stroop test with five topics identified topics referring to “test,” “patient,” “function,” “execut,” “memori,” “age,” and “impair.” However, different fields were grouped together in the same topic (Supplementary Table 5). Moreover, it is possible that some potential fields, such as aging and neuroscience, were covered by LDA for visual search (Supplementary Table 6). In contrast, it is difficult to interpret the results of topic modeling with 50 or more topics because of the large number of topics and their contents (Supplementary Tables 7, 8, 9, and 10). Therefore, we selected LDA with 20 topics with the following results.

#### LDA Topic Modeling for the Abstracts of the Stroop Test

Table 1 shows the top five probable words for each topic of modeling for the manuscript abstracts related to the Stroop test. Topics 2, 5, 17, and 18 consisted of basic words related to the Stroop test experiments, such as “word,” “color,” “process,” “experi,” “respons,” “conflict,” “control,” and “task.” Therefore, these topics clearly represent basic research on the Stroop test. Topics 7 and 10 consisted of words referring to clinical studies, such as “patient,” “impair,” “depress,” and “schizophrenia.” Topics 13 and 19 consisted of words related to aging and developmental studies, such as “age,” “children,” “adult,” and “older.” Finally, Topic 20 consisted of words related to neuroscience, such as “active,” “brain,” and “cortex.”

**Table 1:** Top five probable words in each topic from topic modeling of the manuscript abstracts related to the Stroop test.

	Top five probable words in each topic				
Topic 1	differ	studi	effect	found	number
Topic 2	word	color	interfer	name	read
Topic 3	model	research	report	self	find
Topic 4	test	function	memori	execut	verbal
Topic 5	process	experi	effect	task	inform
Topic 6	subject	time	test	perform	reaction
Topic 7	patient	cognit	impair	test	diseas
Topic 8	relat	use	alcohol	depend	attent
Topic 9	use	measur	analysi	data	score
Topic 10	patient	depress	group	disord	symptom
Topic 11	stress	dure	respons	increas	mental
Topic 12	exercis	cognit	train	group	effect
Topic 13	group	age	inhibit	children	control
Topic 14	treatment	cognit	effect	improv	chang
Topic 15	emot	relat	negat	anxieti	posit
Topic 16	associ	cognit	age	level	year
Topic 17	respons	conflict	trial	task	incongru
Topic 18	control	task	attent	cognit	select
Topic 19	task	perform	adult	older	condit
Topic 20	activ	brain	function	cortex	region

#### LDA Topic Modeling for the Abstracts of Visual Search

Table 2 shows the top five probable words for each topic of modeling for the manuscript abstracts related to visual search. Topics 7, 12, 17, and 19 consisted of basic words related to visual search experiments, such as “target,” “trial,” “distractor,” “featur,” “time,” “display,” “attent,” and “select.” Therefore, these topics clearly represent basic research of visual search. Topic 10 consisted of words referring to clinical studies, such as “patient,” “deficit,” “neglect,” and “field,” which probably refer to unilateral spatial neglect (e.g., Brain, 1941), with a visual search task. Topic 4 consisted of words related to aging and developmental studies, such as “age” and “children.” Furthermore, Topics 14 and 18 consisted of words related to neuroscience, such as “active,” “area,” “region,” “cortex,” “eye,” “movement,” and “saccade.”

**Table 2:** Top five probable words in each topic from topic modeling of the manuscript abstracts related to visual search.

	Top five probable words in each topic				
Topic 1	task	memori	work	perform	load
Topic 2	respons	process	relat	stimulus	compon
Topic 3	orient	contrast	motion	line	differ
Topic 4	age	cognit	group	adult	children
Topic 5	cue	spatial	learn	effect	reward
Topic 6	face	detect	stimuli	experi	emot
Topic 7	target	trial	locat	present	one
Topic 8	perform	effect	train	improv	task
Topic 9	imag	observ	error	rate	detect
Topic 10	patient	control	impair	deficit	neglect
Topic 11	object	item	scene	inform	represent
Topic 12	target	distractor	featur	color	conjunct
Topic 13	high	particip	bias	task	low
Topic 14	activ	area	region	neural	dure
Topic 15	process	perceptu	find	percept	mechan
Topic 16	use	vision	studi	drive	driver
Topic 17	time	display	size	set	condit
Topic 18	eye	movement	fixat	saccad	dure
Topic 19	attent	select	captur	top	irrelev
Topic 20	model	predict	data	use	human

Taken together, we showed that LDA topic modeling with the abstracts of papers related to the Stroop test and visual search identified not only the topics referring to basic contents of the psychological experiments but also those referring to clinical, aging, developmental, and neuroscience research.

### Study 2

**Figure 2:**
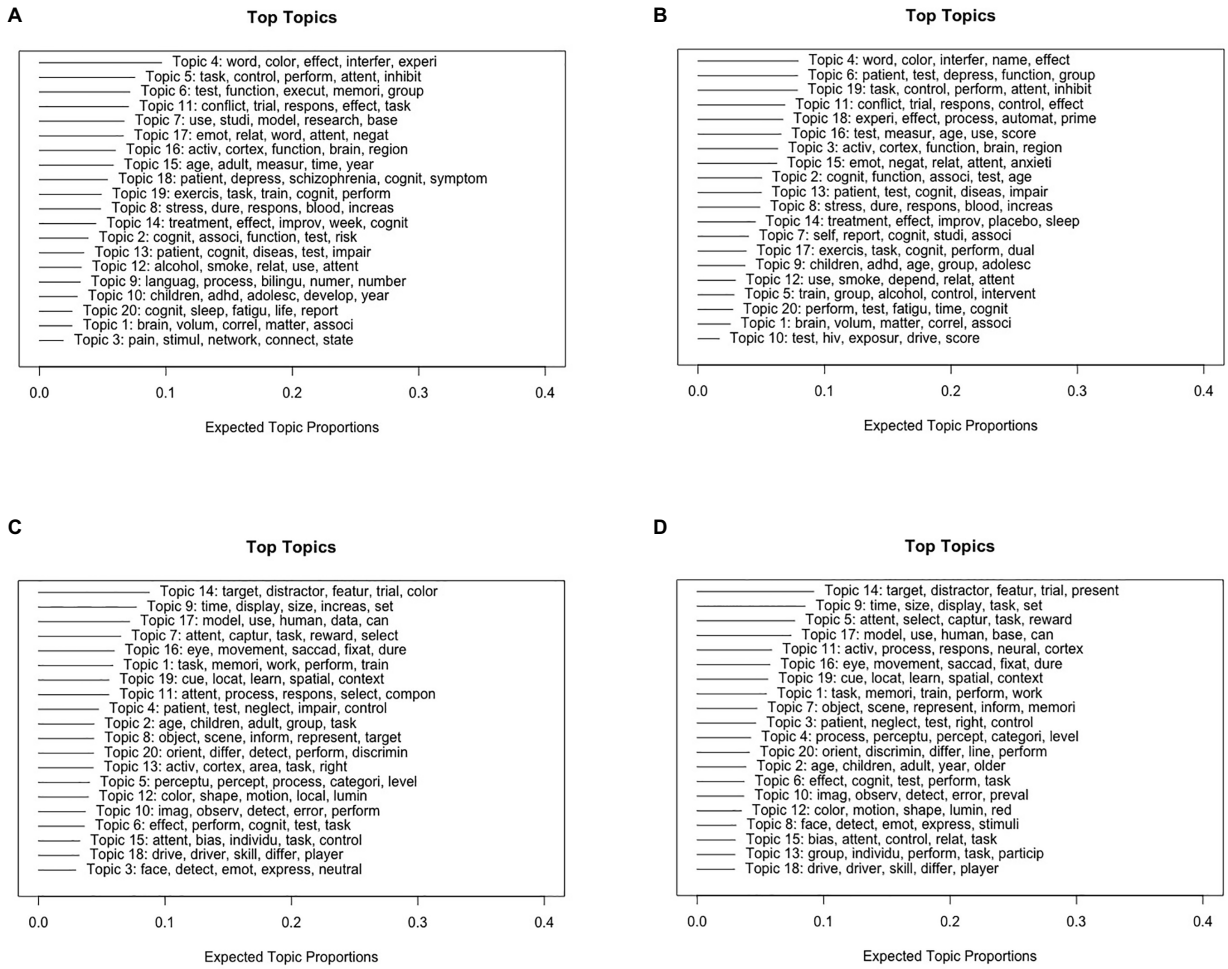
Top five probable words in each topic and expected topic proportions in the STM. **(A)** Top five probable words in each topic and expected topic proportions with three geographic regions (North-Central America, Europe, and Asia) as metadata of STM of the manuscript abstracts related to the Stroop test. **(B)** Top five probable words in each topic and expected topic proportions with publication periods as metadata of STM of the manuscript abstracts related to the Stroop test. **(C)** Top five probable words in each topic and expected topic proportions with three geographic regions (North-Central America, Europe, and Asia) as metadata of STM of the manuscript abstracts related to visual search. **(D)** Top five probable words in each topic and expected topic proportions with publication periods as metadata of STM of the manuscript abstracts related to visual search.

Similar to Study 1, it is difficult to interpret the results of STM with 80 topics for the Stroop test and 70 topics for visual search because of the large number of topics and their contents (Supplementary Figures 5, 6). Therefore, we selected STM with 20 topics with the following results. Figure 2 shows the top five probable words for each topic of STM with three geographic regions and publication periods as the metadata of STM, respectively.

#### STM for the Abstracts of the Stroop Test and Visual Search With Three Geographic Regions as Metadata

**Figure 3:**
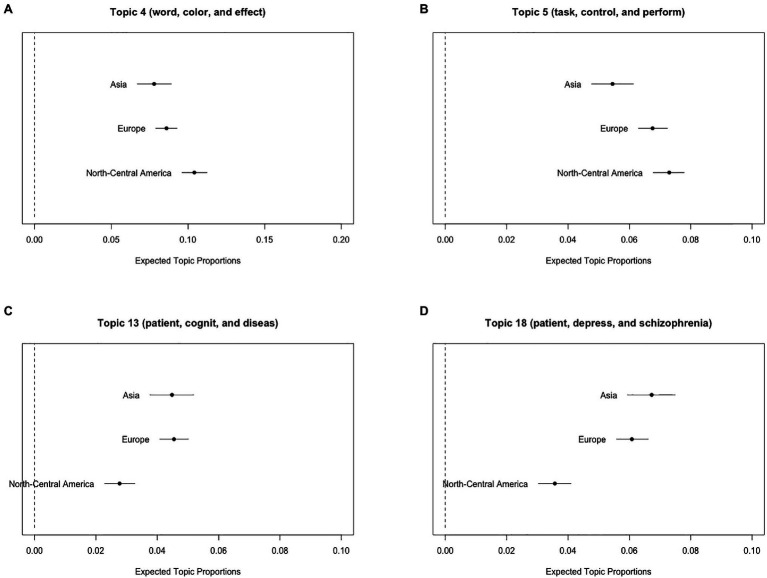
Graphical displays of expected topic proportions and 95% confidence intervals of the mean proportions from the STM of the manuscript abstracts related to the Stroop test as a function of three geographic regions (North-Central America, Europe, and Asia). **(A)** Topic 4 (“word,” “color,” and “effect”), **(B)** Topic 5 (“task,” “control,” and “perform”), **(C)** Topic 13 (“patient,” “cognit,” and “diseas”), and **(D)** Topic 18 (“patient,” “depress,” and “schizophrenia”).

First, we describe the results of the Stroop test with three geographic regions as the metadata of STM. Figures 3A,B illustrate the expected topic proportions of Topics 4 (e.g., “word,” “color,” and “effect”) and 5 (e.g., “task,” “control,” and “perform”) related to the Stroop test experiments as a function of the three geographic regions (i.e., North-Central America, Europe, and Asia). These results showed that the topic proportions referring to the Stroop test experiments differed among the three geographic regions. Specifically, Topic 4 consisting of “word,” “color,” and “effect” was more likely to appear in North-Central America than in Europe [the results of the function *estimateEffect*: *t*(7011) = 3.49, *p* < 0.001] and Asia [*t*(7011) = 4.01, *p* < 0.001]. On the other hand, Topics 13 and 18 related to clinical research, such as “patient,” “cognit,” “diseas,” “depress,” and “schizophrenia,” were more likely to appear in Europe and Asia than in North-Central America [between Europe and North-Central America: *t*(7011) = 4.95, *p* < 0.001 in Topic 13, and *t*(7011) = 6.17, *p* < 0.001 in Topic 18; between Asia and North-Central America: *t*(7011) = 3.90, *p* < 0.001 in Topic 13, and *t*(7011) = 6.25, *p* < 0.001 in Topic 18; Figures 3C,D].

**Figure 4:**
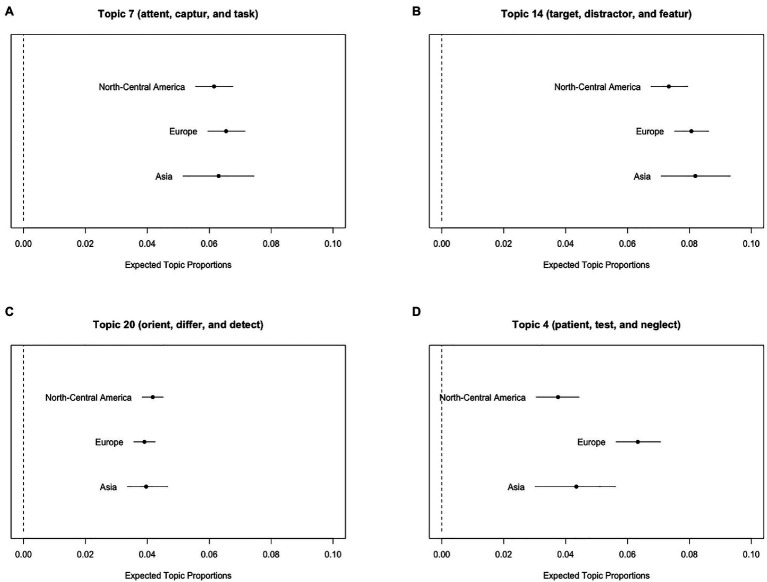
Graphical displays of expected topic proportions and 95% confidence intervals of the mean proportions from the STM of the manuscript abstracts related to visual search as a function of three geographic regions (North-Central America, Europe, and Asia). **(A)** Topic 7 (“attent,” “captur,” and “task”), **(B)** Topic 14 (“target,” “distractor,” and “featur”), **(C)** Topic 20 (“orient,” “differ,” and “detect”), and **(D)** Topic 4 (“patient,” “test,” and “neglect”).

Next, we describe the results of visual search with three geographic regions as the metadata of STM. Figures 4A–C illustrate the expected topic proportions of Topics 7, 14, and 20 as a function of the three geographic regions. These results suggest that the expected topic proportions related to visual search experiments, consisting of “attent,” “captur,” “target,” “distractor,” “orient,” and “differ,” did not differ among the geographic regions. In contrast, the expected proportions of Topic 4 related to clinical research, consisting of “patient,” “test,” and “neglect,” differed among the three geographic regions (Figure 4D), and this topic was more likely to appear in Europe than in North-Central America and Asia [*t*(5149) = 4.90, *p* < 0.001 and *t*(5149) = 2.66, *p* = 0.008, respectively].

Overall, we observed that the expected proportions of Topic 4 related to the Stroop test experiments were high in North-Central America, whereas topics related to visual search experiments did not exhibit regional differences. Moreover, topics related to clinical research were high in Europe irrespective of whether they were related to the Stroop test or visual search. More importantly, the pattern of the main results using the Stroop test abstracts was observed on both STM with 80 topics (Supplementary Figure 7) and STM with 20 topics and two geographic regions (North-Central America: 2,551 abstracts vs. Europe: 2,551 abstracts, which were randomly sampled from 3,073 abstracts to equate the size; Supplementary Figure 8). Moreover, it was true for the results of visual search in both STM with 70 topics (Supplementary Figure 9) and STM with 20 topics and two geographic regions (North-Central America: 2,245 abstracts vs. Europe: 2,245 abstracts, randomly sampled from 2,274 abstracts to equate the size; Supplementary Figure 10).

#### Frequency Analysis of Keywords Among the Three Geographic Regions

In this section, we examined whether keywords of manuscripts show the same patterns of regional differences as those found using STM for the abstracts. We calculated the frequencies and percentages of keywords of manuscripts related to the Stroop test and visual search among the three geographic regions (North-Central America, Europe, and Asia; Supplementary Tables 11 and 12). Because no characteristic differences in percentages of the top 10 probable keywords were found among the three geographic regions, we focused on the frequencies of keywords corresponding to the top 10 probable words belonging to notable topics of the Stroop test and visual search observed in the previous section.

Table 3 shows the total frequencies of keywords of each manuscript corresponding to the top 10 probable words of Topics 4, 13, and 18 of the Stroop test and Topics 4, 7, and 14 of visual search, as a function of the three geographic regions. As a result of the chi-square test (*χ*^2^ (4) = 12.09, *p* = 0.017, *N* = 10,748, Cramer’s *V* = 0.02) and residual analysis for keywords of the Stroop test, the frequencies of keywords corresponding to the words of Topic 4 reflecting the Stroop test experiments were significantly lower than the expected value (809.21) in North-Central America (*p* = 0.015) and significantly higher than the expected value (1162.62) in Europe (*p* = 0.002). Furthermore, the frequencies of keywords corresponding to the words of Topic 18 reflecting clinical research were significantly lower than the expected value (1931.39) in Europe (*p* = 0.012). Moreover, as a result of the chi-square test (*χ*^2^ (4) = 15.45, *p* = 0.004, *N* = 4,898, Cramer’s *V* = 0.04) and residual analysis for keywords of visual search, the frequencies of keywords corresponding to the words of Topic 4 indicating clinical research were marginally higher than the expected value (477.37) in Europe (*p* = 0.081). The frequencies of keywords corresponding to the words of Topic 14 indicating visual search experiments were significantly lower than the expected value (403.16) in Europe (*p* = 0.004).

**Table 3:** Total frequencies of manuscript keywords corresponding to the top 10 probable words of Topics 4, 13, and 18 of the Stroop test and Topics 4, 7, and 14 of visual search from the structural topic modeling, as a function of three geographic regions (North-Central America, Europe, and Asia).

Stroop test			
	North-Central America	Europe	Asia
Topic 4	759	1,232	586
Topic 13	1,239	1,749	902
Topic 18	1,377	1,868	1,036
Visual search			
	North-Central America	Europe	Asia
Topic 4	390	502	118
Topic 7	1,215	1,448	372
Topic 14	349	365	139

The numbers of manuscripts with keywords were 5,606 in the Stroop test and 3,491 in visual search. The total numbers of keyword occurrences of the Stroop test in each region were as follows: North-Central America: 15,346; Europe: 20,706; Asia: 10,327. The total numbers of keyword occurrences of visual search in each region were as follows: North-Central America: 10,378; Europe: 12,426; Asia: 3,637.

Overall, the frequencies of keywords corresponding to clinical research were low in Europe, and those corresponding to the Stroop test experiments were low in North-Central America. Thus, the frequency of keywords cannot capture the trends in STM of the manuscript abstracts.

#### STM for the Abstracts of the Stroop Test and Visual Search With Publication Periods as Metadata

**Figure 5:**
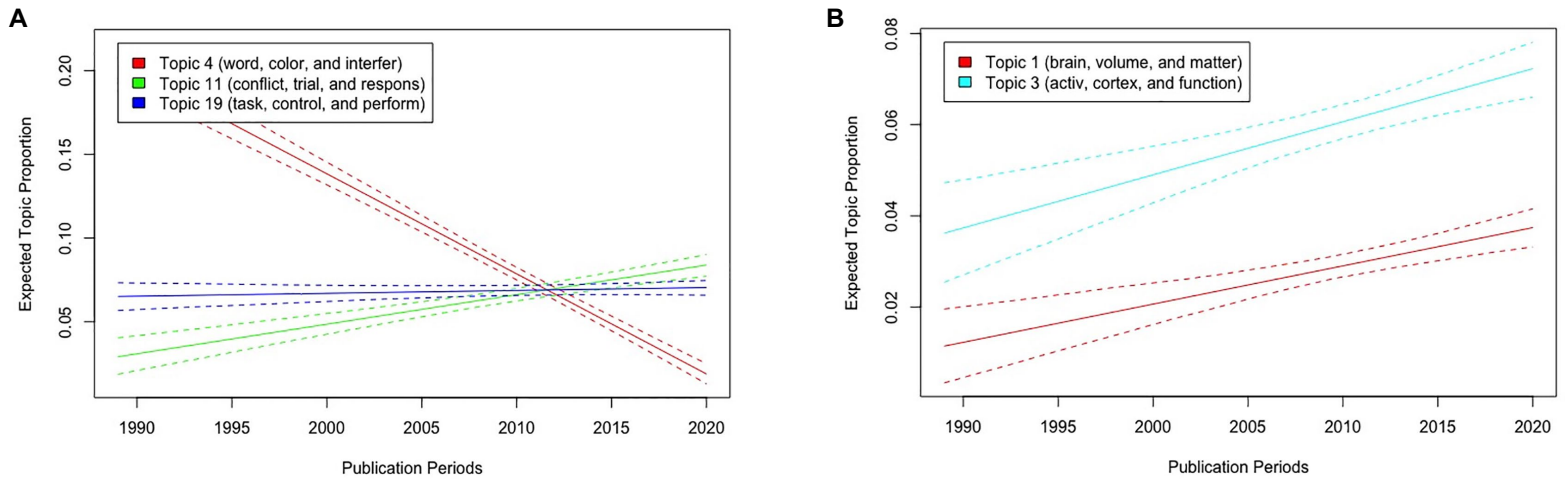
Graphical displays of expected topic proportions and 95% confidence intervals of the STM of the manuscript abstracts related to the Stroop test with publication periods as metadata of STM. **(A)** Proportions of Topics 4 (“word,” “color,” and “interfer”), 11 (“conflict,” “trial,” and “respons”), and 19 (“task,” “control,” and “perform”) as a function of publication periods. **(B)** Proportions of Topics 1 (“brain,” “volume,” and “matter”) and 3 (“activ,” “cortex,” and “function”) as a function of publication periods.

First, we describe the results of the Stroop test with publication periods as the metadata of STM. Figure 5A illustrates the expected topic proportions of Topics 4, 11, and 19, which consisted of basic words related to the Stroop test experiments as a function of publication periods. The results showed that while the expected proportion of Topic 4 (“word,” “color,” and “interfer”) has decreased as a function of publication periods (the results of the function *estimateEffect*: *t*(7506) = −20.33, *p* < 0.001), that of Topic 11 (“conflict,” “trial,” and “respons”) has increased [*t*(7506) = 6.89, *p* < 0.001]. In contrast, the proportion of Topic 19 (“task,” “control,” and “perform”) did not differ across the publication periods [*t*(7506) = 0.766, *p* = 0.444]. Furthermore, the proportions of both Topics 1 (“brain,” “volume,” and “matter”) and 3 (“activ,” “cortex,” and “function”), which consisted of words related to neuroscience, have increased as a function of publication periods [*t*(7506) = 4.81, *p* < 0.001 and *t*(7506) = 4.47, *p* < 0.001, respectively; Figure 5B].

**Figure 6:**
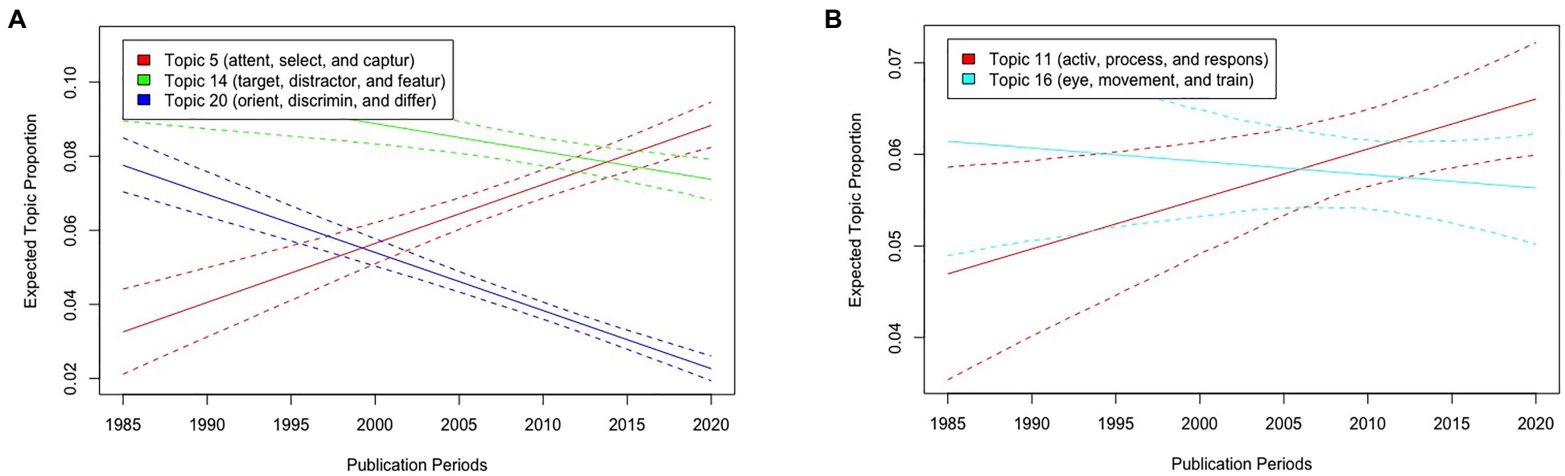
Graphical displays of expected topic proportions and 95% confidence intervals of the STM of the manuscript abstracts related to visual search with publication periods as metadata of STM. **(A)** Proportions of Topics 5 (“attent,” “select,” and “captur”), 14 (“target,” “distractor,” and “feature”), and 20 (“orient,” “discrimin,” and “differ”) as a function of publication periods. **(B)** Proportions of Topics 11 (“activ,” “process,” and “respons”) and 16 (“eye,” “movement,” and “train”) as a function of publication periods.

Next, we describe the results of visual search with publication periods as the metadata of STM. Figure 6A illustrates the expected topic proportions of Topics 5, 14, and 20, which consisted of basic words related to visual search experiments as a function of publication periods. The results showed that while the expected proportions of Topics 14 (“target,” “distractor,” and “featur”) and 20 (“orient,” “discrmin,” and “differ”) have decreased as a function of publication periods [*t*(5406) = −3.50, *p* < 0.001, and *t*(5406) = −11.68, *p* < 0.001], those of Topic 5 (“attent,” “select,” and “captur”) have increased [*t*(5406) = 7.28, *p* < 0.001]. Moreover, the expected proportions of Topic 16 (“eye,” “movement,” and “train”) did not differ across publication periods [*t*(5406) = −0.623, *p* = 0.533], whereas those of Topic 11 (“activ,” “process,” and “respons”) have increased [*t*(5406) = 2.42, *p* = 0.015; Figure 6B].

Taken together, although the trends of the topic proportions related to the Stroop test and visual search experiments differed depending on the contents of observed topics, the proportions of topics related to neuroscience have increased as a function of the publication periods. Again, the pattern of the main results using the Stroop test abstracts was observed on both STM with 80 topics (Supplementary Figure 11) and STM with 20 topics and down-sampling of the number of abstracts in each publication period to 218, which is the minimum among the publication periods (1995–1997; Supplementary Figure 12). Moreover, it was true for the results of visual search on both STM with 70 topics (Supplementary Figure 13) and STM with 20 topics and down-sampling of the number of abstracts in each publication period to 203, which is the minimum among the publication periods (both 1986–1992 and 1997–1999; Supplementary Figure 14). For the results of STM excluding the manuscripts published before 1990 (because the retrieved abstracts before 1990 were sparse), those of interactions between the geographic regions and publication periods, and those of non-linear trends, see Supplementary Material and Supplementary Figures 15–20.

#### STM for the Abstracts of Manuscripts Published in Psychological Science From 1990 to 2020

Although we observed hot topics related to neuroscience research for both the Stroop test and visual search abstracts, some might wonder whether these trends are due to an increase in the number of accepted papers with neuroscience approach in a given journal. To examine this issue, we conducted an additional STM for the abstracts of manuscripts published in *Psychological Science* from 1990 to 2020, with publication periods as metadata of STM. We selected *Psychological Science* as a target journal for examination because it is a general-interest and influential journal in psychology. We obtained 4,112 abstracts of manuscripts published in *Psychological Science* and the corresponding publication years from Scopus database on February 15, 2021. They did not include Erratum, Corrigendum, Reply, Commentary, Retraction, and Editorial items. We conducted the same preprocessing as in Study 1. Again, we created nine publication periods so that each period had the number of abstracts as close to 200 as possible: before 1994 was defined as 1993 (206 abstracts), 1994–1997 as 1997 (256 abstracts), 1998–2000 as 2000 (265 abstracts), 2001–2003 as 2003 (292 abstracts), 2004–2005 as 2005 (294 abstracts), 2006–2007 as 2007 (326 abstracts), 2015–2016 as 2016 (328 abstracts), 2017–2018 as 2018 (312 abstracts), 2019–2020 as 2020 (262 abstracts). We used each publication year from 2008 to 2014 separately (Supplementary Figure 21). We conducted STM with the same parameters as the STM conducted with publication periods as metadata for the Stroop test and visual search.

**Figure 7:**
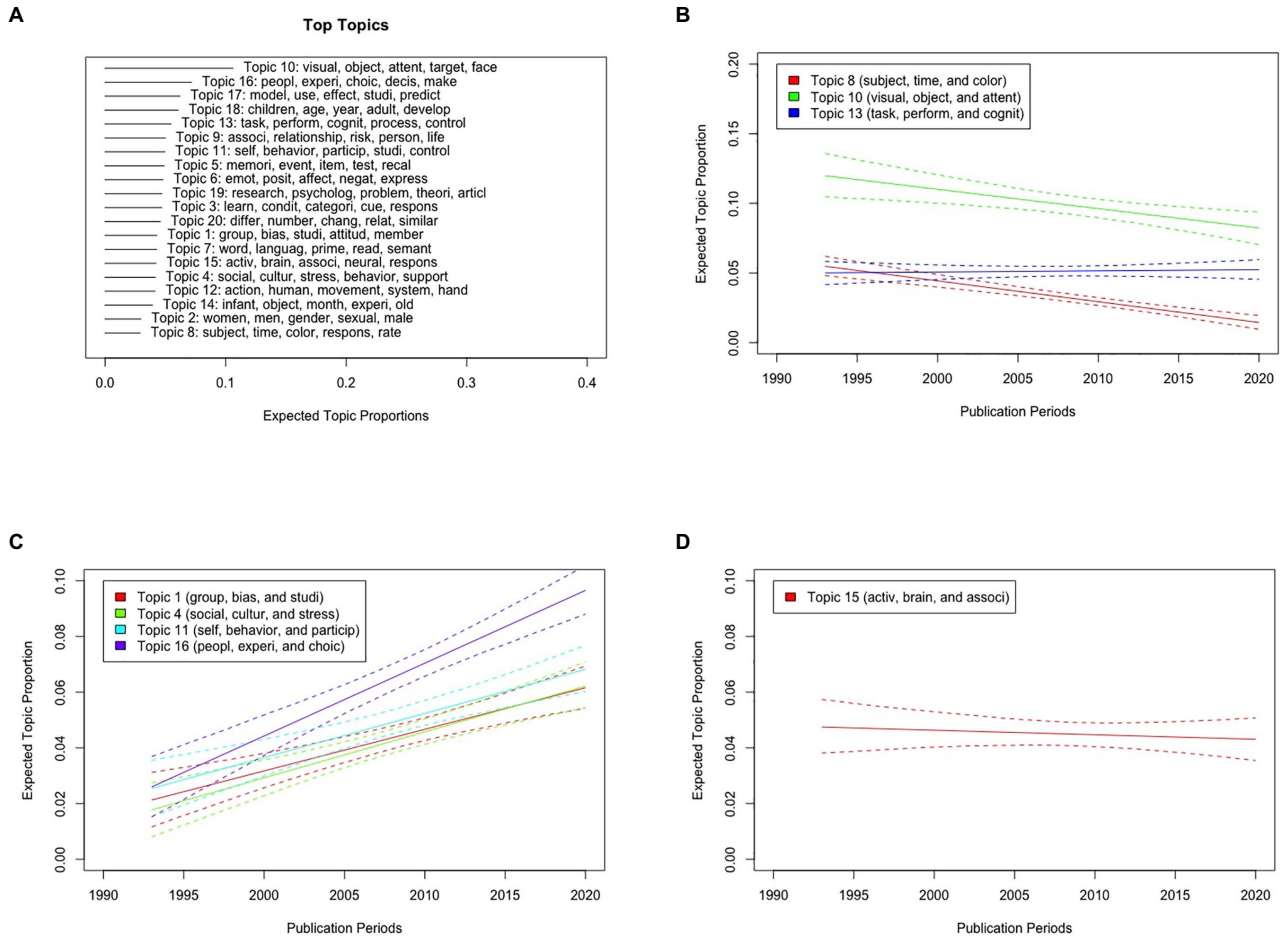
Results of the STM for abstracts of manuscripts published in *Psychological Science* from 1990 to 2020 with publication periods as metadata of STM. **(A)** Top five probable words in each topic and expected topic proportions in STM. **(B)** Graphical display of the expected topic proportions and 95% confidence intervals of Topics 8 (“subject,” “time,” and “color”), 10 (“visual,” “object,” and “attent”), and 13 (“task,” “perform,” and “cognit”) as a function of publication periods. **(C)** Graphical display of the expected topic proportions and 95% confidence intervals of Topics 1 (“group,” “bias,” and “studi”), 4 (“social,” “cultur,” and “stress”), 11 (“self,” “behavior,” and “particip”), and 16 (“peopl,” “experi,” and “choic”) as a function of publication periods. **(D)** Graphical display of the expected topic proportions and 95% confidence intervals of Topic 15 (“activ,” “brain,” and “associ”) as a function of publication periods.

Figure 7A shows the top five probable words in each topic and expected topic proportions of STM. STM for the abstracts of manuscripts published in *Psychological Science* showed various topics reflecting visual attention, social decision, aging and developmental studies, memory, emotion, cultural psychology, personality, gender, language, and neuroscience. This indicates on a probabilistic model that *Psychological Science* is a general-interest journal. In addition, the proportions of Topics 8 (“subject,” “time,” and “color”) and 10 (“visual,” “object,” and “attent”) reflecting human visual attention and cognition have decreased as a function of publication periods [*t*(4110) = −7.58, *p* < 0.001 and *t*(4110) = −3.05, *p* = 0.002, respectively; Figure 7B], whereas those of Topics 1 (“group,” “bias,” and “studi”), 4 (“social,” “cultur,” and “stress”), 11 (“self,” “behavior,” and “particip”), and 16 (“peopl,” “experi,” and “choic”) reflecting cultural and social psychology have increased over the years [*t*(4110) = 5.33, *p* < 0.001, *t*(4110) = 5.50, *p* < 0.001, *t*(4110) = 5.24, *p* < 0.001, and *t*(4110) = 8.14, *p* < 0.001, respectively; Figure 7C]. More importantly, the STM showed that the proportions of Topic 15 (“activ,” “brain,” and “associ”) reflecting neuroscience did not differ across publication periods [*t*(4110) = −0.568, *p* = 0.570; Figure 7D]. These results suggest that the number of manuscripts related to neuroscience research has not increased in the investigated psychology journal across publication periods.

## Discussion

First, in Study 1, we observed not only topics consisting of basic words related to experiments involving both the Stroop test and visual search, but also those consisting of words referring to clinical, developmental, aging, and neuroscience research. These results suggest that topic modeling for psychology manuscript abstracts retrieved from a research database, such as PubMed, is effective for overviewing the trends of research themes.

### Diversity of Geographic Regions and Time Trends in Research Activities in Psychology

More importantly, in Study 2, STM for the abstracts related to the Stroop test with geographic regions as metadata showed that the proportions of topics consisting of “word,” “color,” and “effect” were higher in North-Central America than in Europe and Asia, whereas those of other topics related to the Stroop test and visual search experiments did not differ among the three geographic regions. In contrast, the proportions of topics related to clinical research were higher in Europe than in North-Central America, irrespective of the use of the Stroop test or visual search. To the best of our knowledge, this is the first quantitative study to provide evidence of diversity of geographic regions in research activities in psychology. It is possible that the observed regional differences are related to the history of psychology in that region. In the United Kingdom, Charles Myers established the National Institute of Industrial Psychology, which engaged in applied psychology in industrial and clinical sites (Bartlett, 1948). Further, Frederic Bartlett and Kenneth Craik helped establish the Medical Research Council (MRC) Applied Psychology Unit in 1944 (Broadbent, 1970), which is now the MRC Cognition and Brain Sciences Unit. This history may influence the governing bodies of academic societies, psychology courses offered in each affiliation, and the acquisition of research funding, which in turn influences the research work in each region. Alternatively, public policy, such as curriculum and research funding, and individual research interests may have influenced the development of research activities in each region.

Note that the regional differences found in STM for the manuscript abstracts were not observed by comparing the frequencies of the corresponding keywords. This is because the authors may intentionally select more global words in psychology as keywords. In contrast, topic modeling of abstracts reflecting the manuscript research contents could capture regional differences in research activities. We therefore emphasize that topic modeling for manuscript abstracts can capture the implicit characteristics of regional differences in the research activities in psychology that the frequencies of keywords might miss.

Next, STM for abstracts related to the Stroop test with the publication periods as metadata showed that the proportion of topics consisting of “word,” “color,” and “interfer” has decreased as a function of publication periods, whereas that of topics consisting of “conflict,” “trial,” and “respons” has increased. In contrast, the proportion of topics consisting of “task,” “control,” and “perform” did not differ over time. Similarly, STM for the abstracts related to visual search showed both universal features and patterns of diversity. Furthermore, the proportions of topics related to neuroscience have increased as a function of publication periods, both for the Stroop test and visual search. Although these results are consistent with Wang et al. (2016), who indicated that the proportion of topics related to brain research has increased as a function of publication year, this study provides the first quantitative evidence of time trends using STM on psychology abstracts. As described in the Supplementary Materials, we observed significant interactions between the geographic regions and publication periods concerning only the topic of neuroscience of visual search, which exhibited an increasing trend in North-Central America, a decreasing trend in Asia, and remained unchanged in Europe. In contrast, we did not observe significant interactions between those regions and periods for the manuscript abstracts of the Stroop test. Thus, it is difficult to interpret these differences. Considering the metadata of the geographic regions and publication periods, number of topics, and size of abstracts used in our study, interactions using STM must be carefully considered.

More interestingly, this trend was not observed with STM for the abstracts of papers published in *Psychological Science*. Thus, the increasing trend of topics related to neuroscience is not due to an increase in the number of accepted papers related to neuroscience research in a specific journal. Instead, it may be due to advancements in measurements and an increasing number of publication venues. Notably, STM showed a consistent increase in the proportions of topics related to cultural and social psychology as a function of publication periods (Figure 7C), which may reflect recent trends in *Psychological Science*.

This study has the following implications on the issues of cultural differences and WEIRD in psychology. It is possible that research activity in psychology is strongly influenced by both geographic regions and time trends, similar to the manner in which individual-level psychological functions are affected by cultural evolution. Moreover, some research activities in psychology are biased in specific regions, similar to the bias of American undergraduate student participants (Henrich et al., 2010, 2020; Kupferschmidt, 2019). In other words, the tacit knowledge of universality holds neither for human behavior (Henrich et al., 2010) nor research activities on human mental processes. By quantifying commonalities and differences in research activities in psychology using numerous research abstracts, as with cultural studies in human behavior involving large-scale investigations worldwide (Mehr et al., 2019; Awad et al., 2020), we can facilitate discussions on how to improve research education and activities in psychology.

### Limitations of This Study and Future Directions

A limitation of this study is that we only examined regional differences in research activities in psychology in North-Central America, Europe, and Asia because of the paucity of abstracts from South America, Africa, and Oceania. We chose the above three regions to maintain a similar sample size according to the discussions of some previous studies (Hu et al., 2019; He et al., 2020). In addition, we conducted LDA topic modeling and STM using the abstracts of manuscripts limited to the Stroop test and visual search reflecting human attentional and cognitive control. Thus, it remains unclear whether the trends observed in this study are valid for other psychological themes and other geographic regions. Consequently, we should conduct topic modeling using the abstracts of manuscripts that are not limited to a specific region or a psychological theme.

Moreover, we classified the geographic region of each study by the first author’s first affiliation. However, those authors who conducted internationally collaborative studies could be from multiple geographic regions. Furthermore, the first author may be affiliated with multiple affiliations in different regions. We did not consider these issues in the current study. Since the text data used in our study was the abstract of each study, it was impossible to capture from it, any information pertaining to the region where that study was conducted. Therefore, we *approximated* the geographic region in which each study was conducted by considering the first author’s first affiliation as the region of the study. To precisely identify the region where each study was conducted, it is necessary to refer to the text of each manuscript. This would require an in-depth assessment of several thousands of papers and is thus beyond the scope of our study; however, this issue of research across multiple regions should be considered in future studies.

Although we quantitatively demonstrated universal features and diversity in research activities in psychology, the exact mechanism by which they occur remains unclear and should be explored in future research. Specifically, our findings do not necessarily reflect the diversity of psychological education because research abstracts are the outputs of psychological research. Therefore, we need to study other documents, for example, course syllabi in psychology departments. This approach will clarify the relationship between research education and activity in each region. The text mining approach with topic modeling, particularly STM, can potentially elucidate the effects of other meta-information with each study on psychological topics. Using topic modeling with generalized correspondence LDA, Rubin et al. (2017) decoded brain activities with large-scale datasets of the functional brain atlas of human cognition and corresponding abstracts of fMRI studies. By building a large-scale database including the information on participants, affiliations, and research education and activity, such as age, behavioral responses, curriculum and course syllabus, individual research interest, and research funding, we can deal with cultural differences in human behavior as well as regional and time differences in research activities in a unified manner. This will clarify common sociocultural factors or separate factors that specifically influence either one. Furthermore, by conducting multivariate analysis with various data, we can examine the relationship between personal factors, such as an individual research interest, and socioeconomic factors, such as education system and research funding in each region.

## Conclusion

In this study, topic modeling with manuscript abstracts related to the Stroop test and visual search exhibited not only the topics related to each task but also those related to clinical, aging, developmental, and neuroscience research. In addition, STM revealed regional and time variabilities in research activities in psychology. Knowledge of universality and diversity in research activity will significantly aid the promotion of systematic and distinctive research activity and education.

## Data Availability Statement

The raw data supporting the conclusions of this article will be made available by the authors, without undue reservation.

## Author Contributions

All authors developed this research concept and contributed to the research design. SO conducted both Study 1 and Study 2 in discussion with YU and JS. Specifically, data collection and data analysis were performed by SO. All authors interpreted the results. SO drafted the manuscript. All authors read and approved the final version of the manuscript for submission.

## Funding

This work was supported by JSPS KAKENHI (Grant Numbers 19H05736 and 19K14472).

## Conflict of Interest

The authors declare that the research was conducted in the absence of any commercial or financial relationship that could be construed as a potential conflict of interest.

## Publisher’s Note

All claims expressed in this article are solely those of the authors and do not necessarily represent those of their affiliated organizations, or those of the publisher, the editors and the reviewers. Any product that may be evaluated in this article, or claim that may be made by its manufacturer, is not guaranteed or endorsed by the publisher.
