# Supplementary material for: Diversity in Psychological Research Activities: Quantitative Approach With Topic Modeling
Source: Front Psychol. 2021 Dec 16;12:773916. doi: 10.3389/fpsyg.2021.773916 (PMC8716499; doi:10.3389/fpsyg.2021.773916)
Supplement: Supplementary file 1 [file Data_Sheet_1.pdf]

Supplementary Material: Diversity in Psychological Research Activities: Quantitative Approach With Topic Modeling

Sachio Otsuka[1*], Yoshiyuki Ueda[2], Jun Saiki[1]

[1]Graduate School of Human and Environmental Studies, Kyoto University, Kyoto, Japan

[2]Kokoro Research Center, Kyoto University, Kyoto, Japan

*** Correspondence:**
Sachio Otsuka
otsuka.sachio.8a@kyoto-u.ac.jp

Supplementary Results

We also conducted STM including the interaction term between the geographic regions and publication periods for the manuscripts related to the Stroop test and visual search. We used the R package *stminsights* (Schwemmer & Guyt, 2021) to visualize the results of interactions (**Supplementary Figures 18** and **19**) but we did not observe significant interactions on the topics related to the Stroop test experiments ($t(7010) = 1.06$, $p = .287$ on Topic 4: color, word, and effect; $t(7010) = .172$, $p = .864$ on Topic 5: task, control, and perform; $t(7010) = .810$, $p = .418$ on Topic 11: conflict, trial, and respons), clinical research ($t(7010) = .674$, $p = .500$ on Topic 13: patient, diseas, and test; $t(7010) = .172$, $p = .863$ on Topic 18: patient, depress, and cognit), and neuroscience studies ($t(7010) = 1.12$, $p = .264$ on Topic 1: brain, volum, and matter; $t(7010) = .709$, $p = .478$ on Topic 16: activ, cortex, and function). However, we observed a significant interaction between the geographic regions and publication periods for the manuscripts related to visual search experiments ($t(5148) = 2.15$, $p = .031$ on Topic 13: activ, cortex, and area), which indicated an increasing trend in North-Central America, decreasing trend in Asia, and no change in Europe. In contrast, the remaining topics showed no interactions ($t(5148) = 1.62$, $p = .106$ on Topic 4: patient, test, and neglect; $t(5148) = .308$, $p = .758$ on Topic 7: attent, captur, and reward; $t(5148) = 1.88$, $p = .060$ on Topic 14: target, distractor, and feature; $t(5148) = .733$, $p = .464$ on Topic 16: eye, movement, and saccad; $t(5148) = .520$, $p = .603$ on Topic 20: orient, differ, and detect).

Supplementary Reference

Schwemmer, C., & Guyt, J. (2021). stminsights: A 'Shiny' application for inspecting structural topic models. (R package Version 0.4.1) [Computer software]. Retrieved from https://cran.r-project.org/web/packages/stminsights/index.html

Supplementary Table 1. Countries, regions, and number of abstracts of the Stroop test

Country	Region	Number of abstracts
Argentina	South America	8
Armenia	North-Central America	1
Australia	Oceania	228
Austria	Europe	36
Bangladesh	Asia	1
Belarus	Europe	2
Belgium	Europe	159
Botswana	Africa	1
Brazil	South America	180
Bulgaria	Europe	2
Canada	North-Central America	449
Chile	South America	4
China	Asia	411
Colombia	South America	8
Costa Rica	North-Central America	1
Croatia	Europe	7
Cuba	North-Central America	5
Cyprus	Europe	4
Czechia	Europe	27
Denmark	Europe	25
Ecuador	South America	1
Egypt	Africa	3
Finland	Europe	37
France	Europe	279
Germany	Europe	483
Greece	Europe	23
Hong Kong	Asia	50
Hungary	Europe	22
Iceland	Europe	1
India	Asia	69
Indonesia	Asia	2
Iran	Asia	79
Ireland	Europe	26
Israel	Asia	150
Italy	Europe	254
Japan	Asia	250
Jordan	Asia	1
Kenya	Africa	1
Lebanon	Asia	1

Lithuania	Europe	5
Luxembourg	Europe	3
Malaysia	Asia	4
Mexico	North-Central America	15
Morocco	Africa	1
Nepal	Asia	4
Netherlands	Europe	380
New Zealand	Oceania	29
Nigeria	Africa	2
North Macedonia	Europe	2
Norway	Europe	60
Pakistan	Asia	1
Poland	Europe	109
Portugal	Europe	30
Qatar	Asia	2
Romania	Europe	2
Russia	Europe	15
Saudi Arabia	Asia	4
Serbia	Europe	3
Singapore	Asia	9
Slovakia	Europe	14
Slovenia	Europe	6
South Africa	Africa	26
South Korea	Asia	151
Spain	Europe	231
Sri Lanka	Asia	1
Sweden	Europe	100
Switzerland	Europe	80
Taiwan	Asia	60
Thailand	Asia	4
Tunisia	Africa	3
Turkey	Asia	132
UK	Europe	645
United Arab Emirates	Asia	3
USA	North-Central America	2,081

Supplementary Table 2. Countries, regions, and number of abstracts of visual search

Country	Region	Number of abstracts
Argentina	South America	6
Australia	Oceania	206
Austria	Europe	22
Bahrain	Asia	1
Bangladesh	Asia	1
Belgium	Europe	45
Brazil	South America	18
Canada	North-Central America	345
Chile	South America	2
China	Asia	154
Colombia	South America	1
Croatia	Europe	2
Cyprus	Europe	2
Czechia	Europe	1
Denmark	Europe	13
Estonia	Europe	8
Finland	Europe	41
France	Europe	109
Georgia	Europe	2
Germany	Europe	473
Greece	Europe	6
Hong Kong	Asia	40
Hungary	Europe	8
Iceland	Europe	42
India	Asia	17
Iran	Asia	7
Ireland	Europe	14
Israel	Asia	89
Italy	Europe	140
Jamaica	North Central America	1
Japan	Asia	236
Lithuania	Europe	2
Malaysia	Asia	3
Malta	Europe	5
Mexico	North Central America	5
Netherlands	Europe	269
New Zealand	Oceania	19
Nigeria	Africa	1
Norway	Europe	17

Poland	Europe	9
Portugal	Europe	15
Russia	Europe	27
Saudi Arabia	Asia	2
Singapore	Asia	11
South Africa	Africa	1
South Korea	Asia	30
Spain	Europe	51
Sweden	Europe	30
Switzerland	Europe	85
Taiwan	Asia	31
Turkey	Asia	7
UK	Europe	836
United Arab Emirates	Asia	3
Uruguay	South America	3
USA	North Central America	1,894

Supplementary Table 3. Number of manuscripts related to the Stroop test in each year

1962	1	1992	52
1963	1	1993	54
1964	0	1994	52
1965	1	1995	58
1966	2	1996	68
1967	4	1997	92
1968	1	1998	86
1969	2	1999	90
1970	1	2000	120
1971	3	2001	115
1972	1	2002	151
1973	4	2003	145
1974	4	2004	171
1975	6	2005	226
1976	5	2006	226
1977	7	2007	244
1978	6	2008	219
1979	8	2009	258
1980	13	2010	300
1981	16	2011	332
1982	9	2012	407
1983	18	2013	451
1984	16	2014	432
1985	18	2015	428
1986	10	2016	454
1987	16	2017	486
1988	21	2018	488
1989	31	2019	548
1990	33	2020	461
1991	36	Total	7,508

Supplementary Table 4. Number of manuscripts related to visual search in each year

1948	1	1985	14
1949	0	1986	9
1950	0	1987	17
1951	0	1988	21
1952	0	1989	47
1953	0	1990	28
1954	0	1991	37
1955	0	1992	44
1956	1	1993	39
1957	0	1994	65
1958	1	1995	47
1959	1	1996	57
1960	0	1997	57
1961	1	1998	75
1962	4	1999	71
1963	3	2000	101
1964	2	2001	114
1965	0	2002	100
1966	3	2003	105
1967	2	2004	150
1968	6	2005	140
1969	1	2006	155
1970	6	2007	157
1971	8	2008	159
1972	8	2009	201
1973	4	2010	218
1974	6	2011	256
1975	12	2012	229
1976	11	2013	297
1977	10	2014	316
1978	20	2015	276
1979	13	2016	300
1980	13	2017	339
1981	9	2018	310
1982	22	2019	315
1983	15	2020	343
1984	16	Total	5,408

Supplementary Table 5. Top five probable words in each topic from topic modeling with 5 topics of the manuscript abstracts related to the Stroop test

	Top five probable words in each topic				
Topic 1	task	effect	word	color	interfer
Topic 2	test	patient	cognit	function	execut
Topic 3	attent	control	relat	emot	task
Topic 4	perform	cognit	effect	signific	dure
Topic 5	control	activ	function	cognit	brain

Supplementary Table 6. Top five probable words in each topic from topic modeling with 5 topics of the manuscript abstracts related to visual search

	Top five probable words in each topic				
Topic 1	attent	memori	particip	locat	cue
Topic 2	eye	use	movement	fixat	saccad
Topic 3	target	distractor	experi	featur	color
Topic 4	task	perform	test	control	group
Topic 5	process	object	task	respons	select

Supplementary Table 7. Top five probable words in each topic from topic modeling with 50 topics of the manuscript abstracts related to the Stroop test

	Top five probable words in each topic				
Topic 1	measur	predict	variabl	factor	relationship
Topic 2	process	inform	automat	task	relev
Topic 3	anxieti	bias	relat	social	threat
Topic 4	semant	prime	languag	effect	pictur
Topic 5	inhibit	control	respons	inhibitori	task
Topic 6	cognit	perform	impair	function	domain
Topic 7	level	associ	concentr	correl	function
Topic 8	memori	test	verbal	learn	work
Topic 9	interfer	effect	number	size	differ
Topic 10	age	adult	year	older	young
Topic 11	use	model	base	data	can
Topic 12	function	execut	test	card	assess
Topic 13	stimul	left	effect	motor	right
Topic 14	test	use	measur	valid	assess
Topic 15	group	control	differ	signific	compar
Topic 16	patient	test	evalu	month	signific
Topic 17	individu	associ	impuls	behavior	decis
Topic 18	task	dual	condit	walk	perform
Topic 19	perform	fatigu	mental	condit	test
Topic 20	control	self	behavior	particip	reduc
Topic 21	chang	dure	increas	use	activ
Topic 22	conflict	trial	incongru	respons	congruent
Topic 23	children	adhd	adolesc	develop	disord
Topic 24	role	evid	find	support	hypothesi
Topic 25	patient	schizophrenia	deficit	control	healthi
Topic 26	relat	potenti	event	erp	compon
Topic 27	test	digit	score	trail	neuropsycholog
Topic 28	use	depend	smoke	relat	drug
Topic 29	report	pain	measur	self	life
Topic 30	treatment	improv	effect	placebo	week
Topic 31	word	color	name	interfer	read
Topic 32	exercis	train	improv	effect	intervent
Topic 33	high	level	low	exposur	suggest
Topic 34	visual	object	auditori	particip	percept
Topic 35	stress	respons	blood	dure	mental
Topic 36	depress	symptom	disord	scale	ptsd
Topic 37	subject	studi	healthi	signific	perform
Topic 38	term	sleep	long	perform	short

Topic 39	women	femal	food	bodi	male
Topic 40	associ	risk	function	cognit	year
Topic 41	attent	select	studi	present	show
Topic 42	effect	experi	respons	stimulus	spatial
Topic 43	brain	correl	frontal	volum	lobe
Topic 44	two	one	first	experi	type
Topic 45	task	perform	two	differ	demand
Topic 46	particip	alcohol	studi	relat	use
Topic 47	time	reaction	error	condit	respons
Topic 48	patient	diseas	cognit	score	impair
Topic 49	activ	cortex	region	brain	function
Topic 50	emot	negat	posit	affect	stimuli

Supplementary Table 8. Top five probable words in each topic from topic modeling with 80 topics of the manuscript abstracts related to the Stroop test

	Top five probable words in each topic				
Topic 1	function	execut	assess	test	use
Topic 2	food	femal	bodi	male	weight
Topic 3	studi	find	previous	evid	present
Topic 4	model	can	account	paradigm	research
Topic 5	process	inform	speed	automat	number
Topic 6	inhibit	task	inhibitori	control	respons
Topic 7	memori	work	shift	perform	abil
Topic 8	relat	potenti	event	erp	compon
Topic 9	cognit	perform	impair	domain	function
Topic 10	life	cognit	health	report	studi
Topic 11	effect	target	prime	stimulus	irrelev
Topic 12	task	perform	switch	demand	particip
Topic 13	test	verbal	memori	learn	recal
Topic 14	self	report	particip	task	mind
Topic 15	predict	factor	variabl	score	regress
Topic 16	relat	ptsd	word	particip	trauma
Topic 17	word	color	name	read	neutral
Topic 18	visual	object	auditori	percept	particip
Topic 19	semant	word	languag	pictur	bilingu
Topic 20	schizophrenia	test	patient	card	sort
Topic 21	time	reaction	accuraci	longer	mean
Topic 22	subject	signific	healthi	normal	perform
Topic 23	test	digit	trail	make	neuropsycholog
Topic 24	differ	pattern	type	across	two
Topic 25	stimul	effect	session	signific	rtms
Topic 26	term	long	short	studi	durat
Topic 27	use	data	analysi	base	signal
Topic 28	experi	letter	color	like	whether
Topic 29	level	concentr	studi	serum	plasma
Topic 30	task	dual	perform	walk	gait
Topic 31	condit	effect	interact	task	load
Topic 32	use	smoke	depend	drug	impuls
Topic 33	control	compar	proactiv	suggest	mechan
Topic 34	negat	posit	correl	affect	relat
Topic 35	two	first	one	present	second
Topic 36	motor	diseas	earli	parkinson	onset
Topic 37	high	individu	low	suggest	level
Topic 38	train	intervent	improv	effect	group

Topic 39	associ	gene	studi	carrier	genet
Topic 40	conflict	adapt	trial	respons	process
Topic 41	respons	respond	stimulus	task	set
Topic 42	error	perform	number	correct	complet
Topic 43	patient	control	disord	healthi	deficit
Topic 44	stress	rate	dure	heart	respons
Topic 45	measur	test	assess	use	correl
Topic 46	attent	select	process	sustain	focus
Topic 47	group	signific	compar	differ	show
Topic 48	anxieti	social	trait	threat	person
Topic 49	adhd	disord	deficit	control	attent
Topic 50	test	patient	multipl	clinic	score
Topic 51	alcohol	particip	studi	student	relat
Topic 52	associ	cognit	risk	year	adjust
Topic 53	women	decis	make	men	phase
Topic 54	chang	dure	prefront	task	use
Topic 55	depress	symptom	patient	scale	rate
Topic 56	sleep	perform	day	test	hour
Topic 57	mood	exposur	test	effect	perform
Topic 58	patient	test	month	surgeri	neuropsycholog
Topic 59	bias	attent	relat	cue	stimuli
Topic 60	studi	use	research	size	effect
Topic 61	associ	perform	measur	higher	examin
Topic 62	frontal	left	right	gyrus	lobe
Topic 63	blood	pressur	mental	stress	dure
Topic 64	cognit	increas	cerebr	perform	function
Topic 65	non	may	studi	compar	histori
Topic 66	treatment	effect	placebo	improv	week
Topic 67	trial	incongru	effect	congruent	congruenc
Topic 68	fatigu	perform	mental	test	dure
Topic 69	behavior	role	process	play	import
Topic 70	children	adolesc	year	age	develop
Topic 71	exercis	physic	acut	fit	intens
Topic 72	pain	chronic	relat	studi	hiv
Topic 73	follow	sever	test	measur	injuri
Topic 74	activ	cortex	brain	region	cingul
Topic 75	interfer	effect	facilit	find	increas
Topic 76	task	postur	balanc	eye	movement
Topic 77	brain	correl	connect	volum	matter
Topic 78	age	adult	older	year	young
Topic 79	patient	cognit	impair	dementia	test

Topic 80	emot	stimuli	face	word	neutral

Supplementary Table 9. Top five probable words in each topic from topic modeling with 50 topics of the manuscript abstracts related to visual search

	Top five probable words in each topic				
Topic 1	memori	work	represent	term	templat
Topic 2	two	one	first	second	multipl
Topic 3	target	distractor	among	similar	present
Topic 4	object	scene	base	natur	real
Topic 5	spatial	frequenc	tempor	present	auditori
Topic 6	model	predict	observ	distribut	account
Topic 7	effect	studi	influenc	factor	affect
Topic 8	item	onset	new	preview	appear
Topic 9	detect	chang	contrast	signal	sensit
Topic 10	time	subject	reaction	rate	increas
Topic 11	perform	measur	speed	accuraci	correl
Topic 12	use	data	system	base	analysi
Topic 13	letter	word	name	semant	particip
Topic 14	size	set	number	increas	slope
Topic 15	research	studi	percept	discuss	cognit
Topic 16	bias	attent	relat	toward	studi
Topic 17	eye	fixat	movement	gaze	dure
Topic 18	trial	target	prime	effect	pop
Topic 19	particip	strategi	environ	perform	instruct
Topic 20	age	children	adult	year	older
Topic 21	condit	high	low	load	level
Topic 22	color	shape	differ	target	red
Topic 23	compon	relat	potenti	npc	event
Topic 24	cognit	test	function	assess	impair
Topic 25	patient	neglect	right	left	impair
Topic 26	experi	effect	present	two	examin
Topic 27	control	group	compar	show	non
Topic 28	learn	context	contextu	repeat	configur
Topic 29	differ	pattern	discrimin	relat	similar
Topic 30	reward	error	associ	task	valu
Topic 31	locat	target	inhibit	probabl	spatial
Topic 32	imag	observ	use	case	radiologist
Topic 33	process	perceptu	stage	percept	inform
Topic 34	activ	cortex	region	area	function
Topic 35	attent	select	shift	mechan	focus
Topic 36	vision	field	drive	driver	peripher
Topic 37	face	detect	emot	stimuli	express

Topic 38	motion	individu	typic	move	asd
Topic 39	human	categori	anim	use	familiar
Topic 40	find	evid	studi	role	hypothesi
Topic 41	train	improv	effect	follow	practic
Topic 42	display	effici	observ	dynam	use
Topic 43	action	skill	decis	make	player
Topic 44	orient	line	local	global	element
Topic 45	respons	stimulus	stimuli	present	array
Topic 46	attent	captur	singleton	irrelev	top
Topic 47	featur	conjunct	target	defin	dimens
Topic 48	cue	inform	use	can	particip
Topic 49	saccad	select	neuron	activ	monkey
Topic 50	task	perform	demand	dual	requir

Supplementary Table 10. Top five probable words in each topic from topic modeling with 70 topics of the manuscript abstracts related to visual search

	Top five probable words in each topic				
Topic 1	memori	work	encod	represent	content
Topic 2	task	perform	demand	difficulti	requir
Topic 3	predict	support	expect	hypothesi	evid
Topic 4	chang	dure	adapt	period	phase
Topic 5	face	express	emot	detect	crowd
Topic 6	activ	cortex	region	function	area
Topic 7	load	capac	perceptu	limit	effect
Topic 8	spatial	tempor	frequenc	auditori	integr
Topic 9	experi	display	effect	two	examin
Topic 10	featur	conjunct	defin	dimens	base
Topic 11	compon	potenti	relat	npc	event
Topic 12	item	guid	templat	match	can
Topic 13	drive	driver	studi	vehicl	simul
Topic 14	reward	associ	valu	previous	influenc
Topic 15	cognit	test	function	assess	measur
Topic 16	use	base	system	data	comput
Topic 17	detect	present	presenc	rapid	suggest
Topic 18	attent	bias	shift	toward	relat
Topic 19	respons	stimulus	present	array	stimuli
Topic 20	perceptu	categori	percept	similar	level
Topic 21	measur	factor	variabl	use	analysi
Topic 22	patient	right	neglect	left	side
Topic 23	activ	neuron	select	neural	respons
Topic 24	effect	perform	test	alcohol	cognit
Topic 25	eye	fixat	movement	gaze	durat
Topic 26	attent	captur	irrelev	distract	relev
Topic 27	target	present	multipl	nontarget	cost
Topic 28	age	adult	relat	year	older
Topic 29	effici	inhibit	previous	studi	ior
Topic 30	imag	radiologist	case	interpret	diagnost
Topic 31	letter	word	semant	name	languag
Topic 32	onset	new	item	preview	appear
Topic 33	train	improv	group	effect	practic
Topic 34	research	studi	review	provid	discuss
Topic 35	patient	control	impair	deficit	healthi
Topic 36	perform	task	improv	better	individu
Topic 37	cue	effect	valid	facilit	present

Topic 38	distractor	target	among	similar	present
Topic 39	subject	use	scan	studi	signific
Topic 40	locat	target	spatial	probabl	appear
Topic 41	trial	prime	effect	target	pop
Topic 42	error	rate	preval	miss	screen
Topic 43	stimuli	posit	negat	threat	emot
Topic 44	skill	player	expert	make	differ
Topic 45	two	one	differ	first	second
Topic 46	local	global	human	structur	anim
Topic 47	group	individu	asd	autism	control
Topic 48	speed	accuraci	identif	slow	use
Topic 49	particip	task	behavior	whether	peopl
Topic 50	report	non	term	long	studi
Topic 51	model	distribut	predict	decis	data
Topic 52	color	differ	experi	red	shape
Topic 53	motion	move	direct	dynam	system
Topic 54	size	set	increas	effect	number
Topic 55	orient	line	element	asymmetri	discrimin
Topic 56	differ	pattern	show	familiar	larg
Topic 57	learn	context	contextu	configur	repeat
Topic 58	field	vision	peripher	central	use
Topic 59	attent	select	mechan	focus	attend
Topic 60	children	read	control	task	deficit
Topic 61	saccad	movement	eye	initi	dure
Topic 62	shape	part	depth	figur	contour
Topic 63	inform	use	action	hand	process
Topic 64	singleton	top	salienc	salient	bottom
Topic 65	process	parallel	serial	stage	interact
Topic 66	time	increas	reaction	number	longer
Topic 67	condit	high	low	level	contrast
Topic 68	object	scene	natur	real	base
Topic 69	observ	strategi	human	use	optim
Topic 70	role	task	involv	studi	play

Supplementary Table 11. Frequencies and percentages of the top 10 probable keywords of manuscripts related to the Stroop test as a function of three geographic regions (North-Central America, Europe, and Asia)

North-Central America			Europe			Asia		
Word	Frequency	Percentage	Word	Frequency	Percentage	Word	Frequency	Percentage
cognit	663	4.32	cognit	878	4.24	cognit	435	4.21
function	402	2.62	function	522	2.52	function	383	3.71
attent	383	2.50	attent	445	2.15	attent	224	2.17
control	322	2.10	task	392	1.89	execut	207	2.00
execut	290	1.89	execut	359	1.73	task	198	1.92
task	216	1.41	control	346	1.67	control	190	1.84
test	183	1.19	test	266	1.28	test	186	1.80
emot	164	1.07	emot	251	1.21	disord	180	1.74
disord	148	0.96	disord	236	1.14	effect	113	1.09
neuropsycholog	148	0.96	interfer	194	0.94	conflict	98	0.95

Supplementary Table 12. Frequencies and percentages of the top 10 probable keywords of manuscripts related to visual search as a function of three geographic regions (North-Central America, Europe, and Asia)

North-Central America			Europe			Asia		
Word	Frequency	Percentage	Word	Frequency	Percentage	Word	Frequency	Percentage
attent	792	7.63	attent	938	7.55	attent	227	6.24
eye	279	2.69	eye	296	2.38	memori	67	1.84
memori	236	2.27	movement	229	1.84	eye	59	1.62
movement	205	1.98	memori	216	1.74	work	41	1.13
percept	145	1.40	cognit	152	1.22	effect	40	1.10
cognit	141	1.36	percept	142	1.14	task	35	0.96
learn	128	1.23	select	128	1.03	cognit	34	0.93
spatial	120	1.16	spatial	125	1.01	learn	33	0.91
select	107	1.03	process	117	0.94	movement	33	0.91
process	100	0.96	work	112	0.90	captur	30	0.82

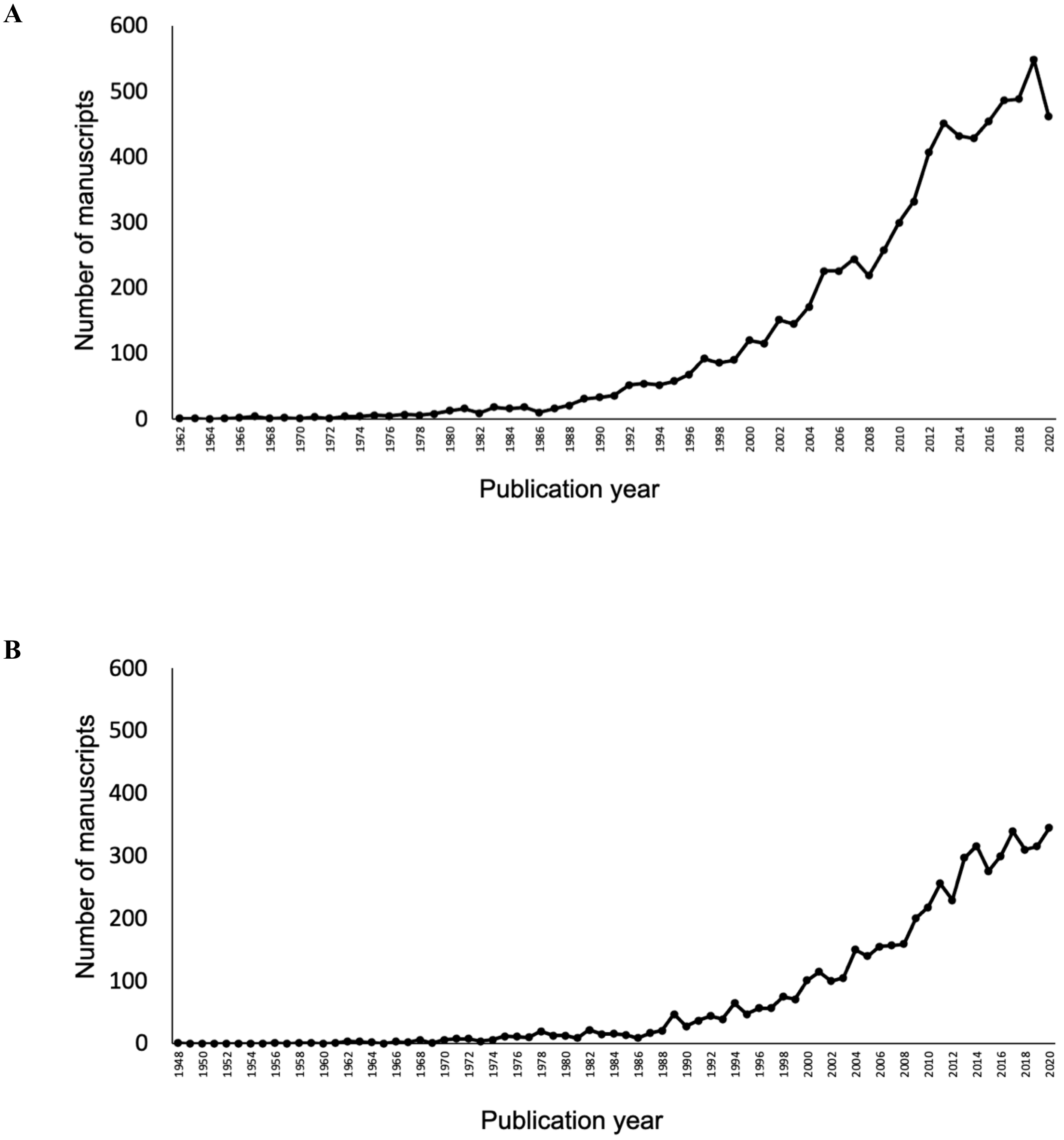

Supplementary Figure 1. Number of manuscripts related to the Stroop test (**A**) and visual search (**B**) as a function of publication year.

A

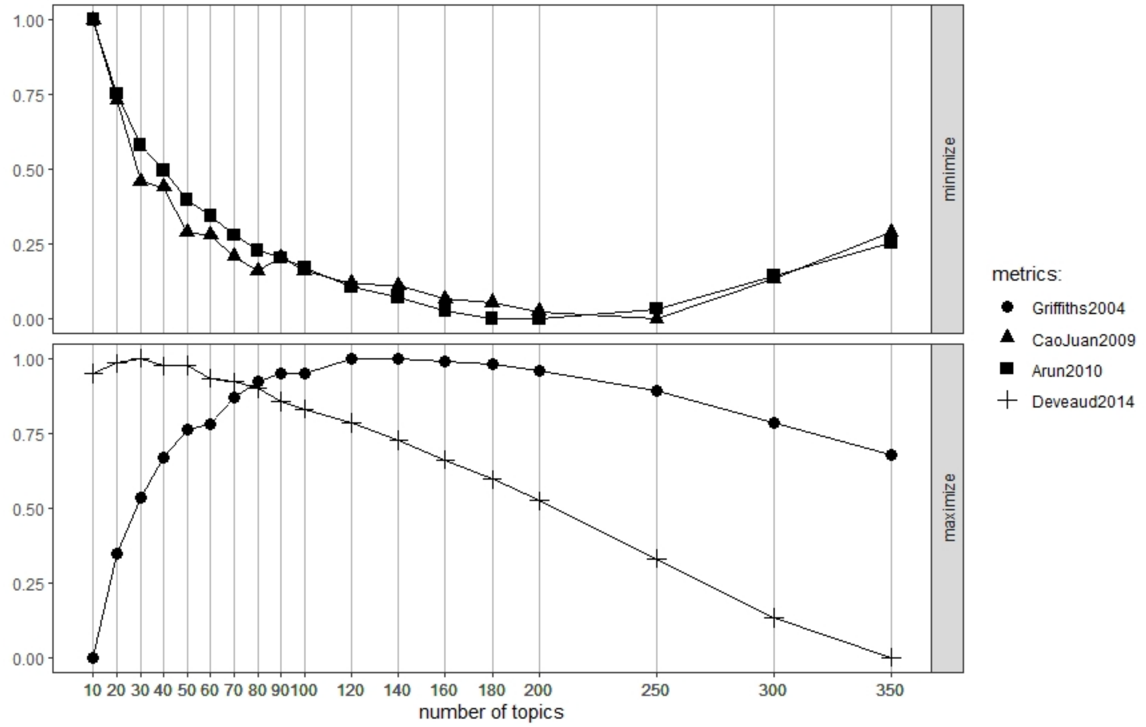

B

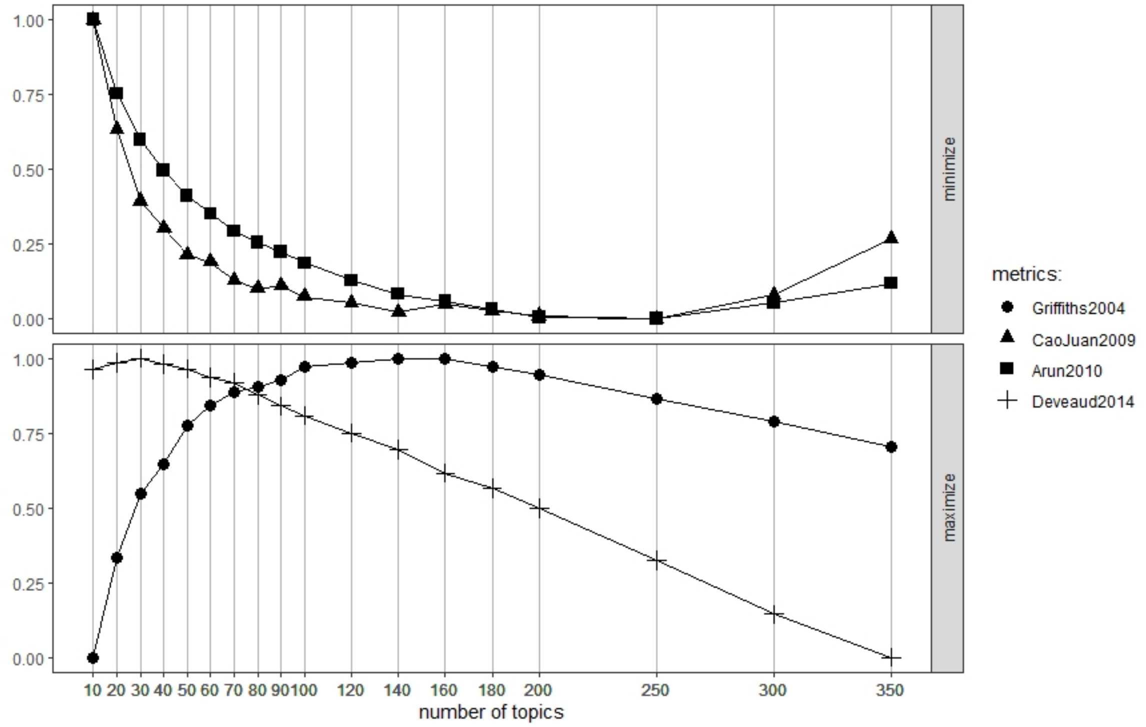

Supplementary Figure 2. Results of *ldatuning* for LDA topic modelling for the manuscripts related to the Stroop test (**A**) and visual search (**B**).

A

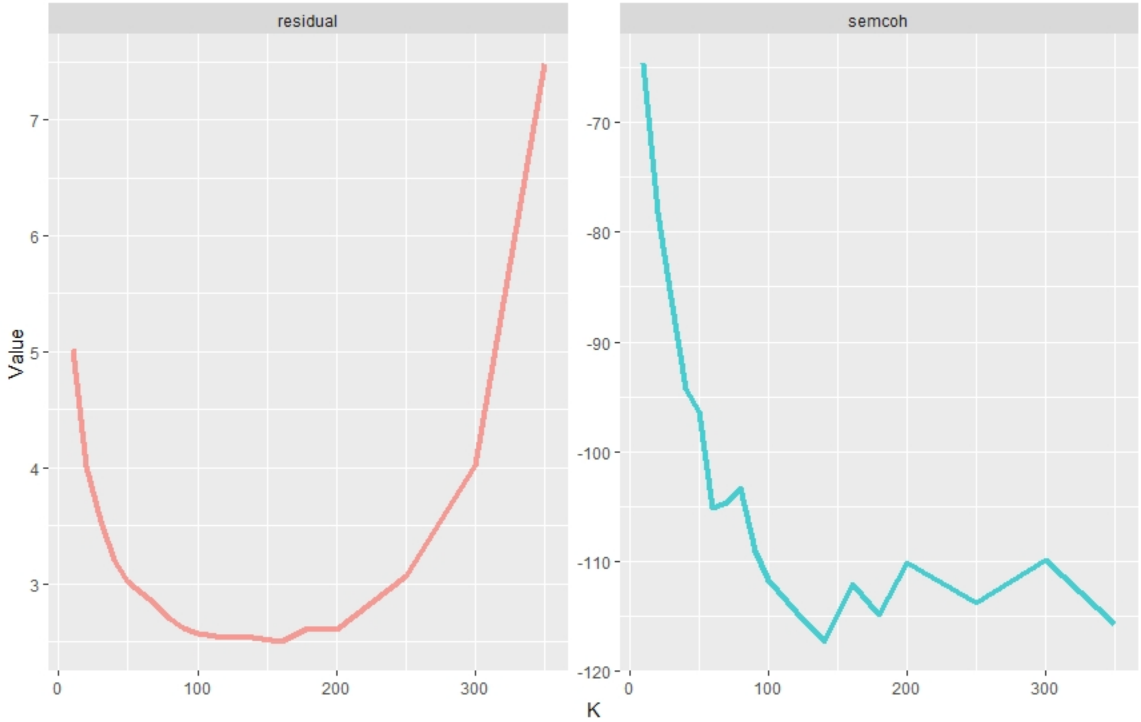

B

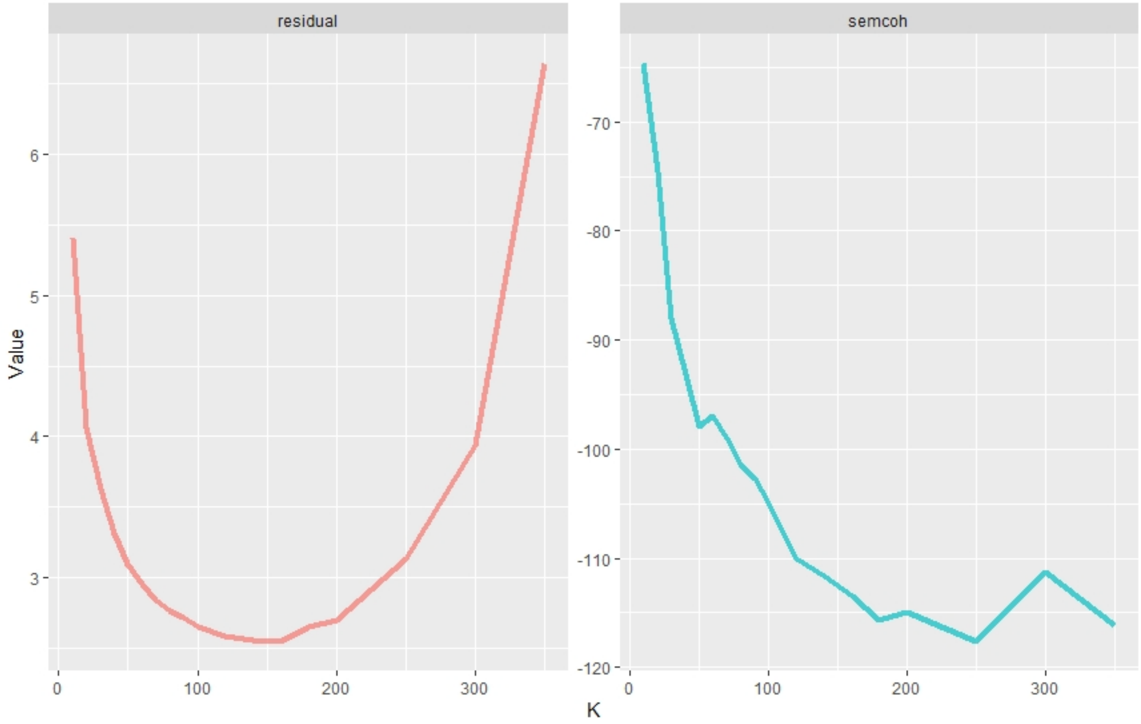

Supplementary Figure 3. Results of *searchK* for the STM of the manuscripts related to the Stroop test with three geographic regions (**A**) and publication periods (**B**) as metadata.

A

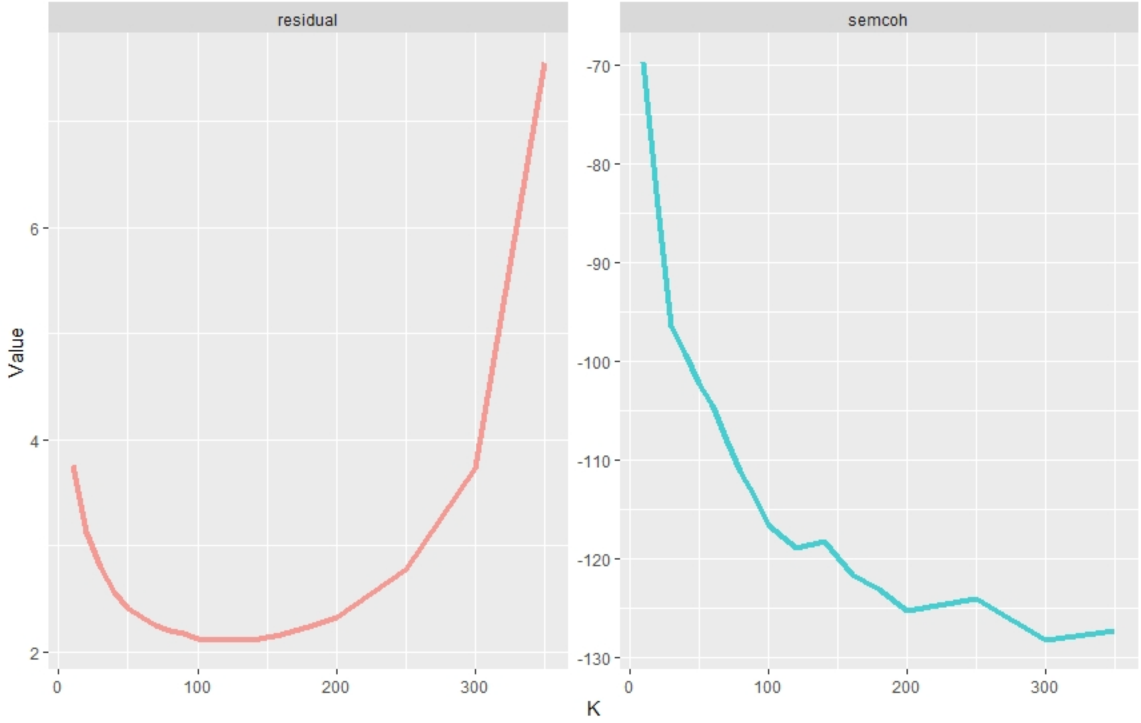

B

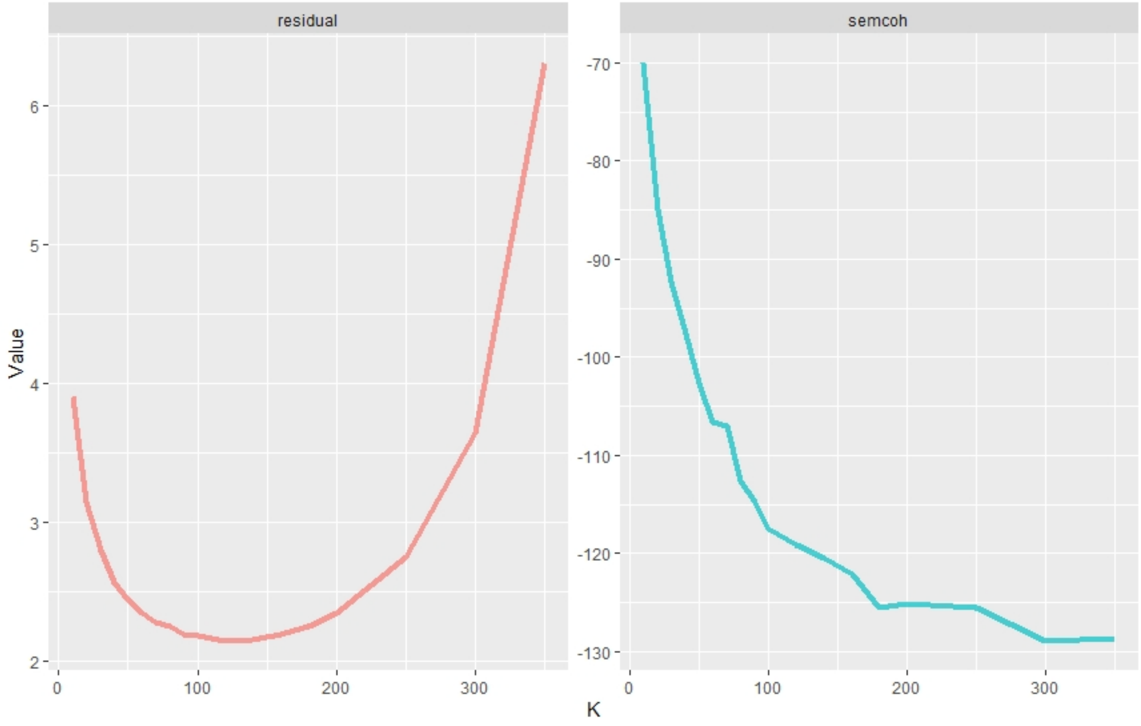

Supplementary Figure 4. Results of *searchK* for the STM of the manuscripts related to visual search with three geographic regions (**A**) and publication periods (**B**) as metadata.

A

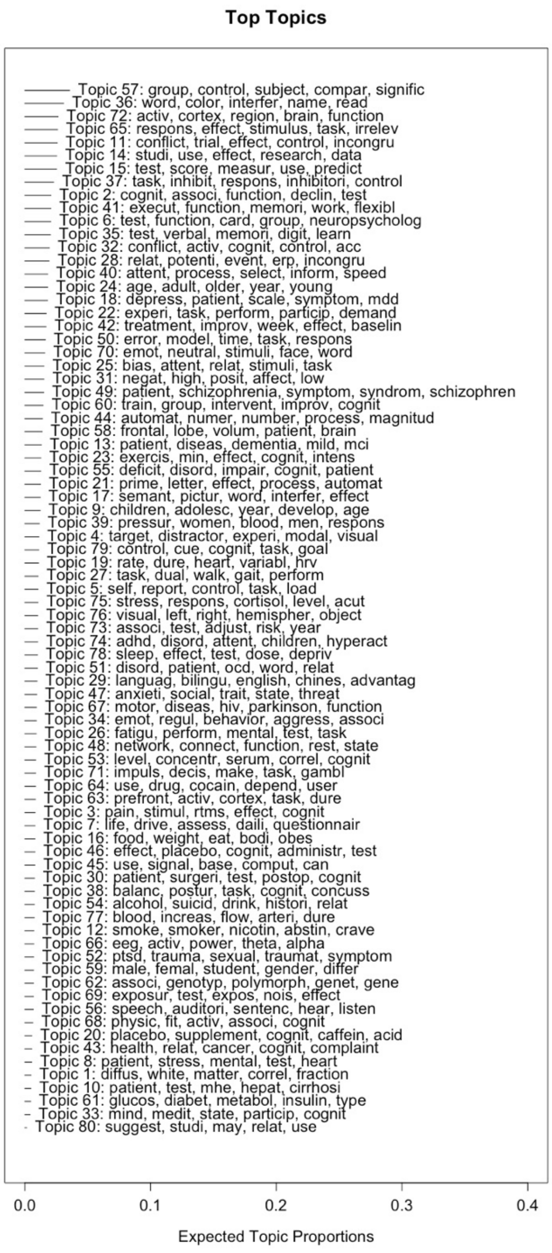

B

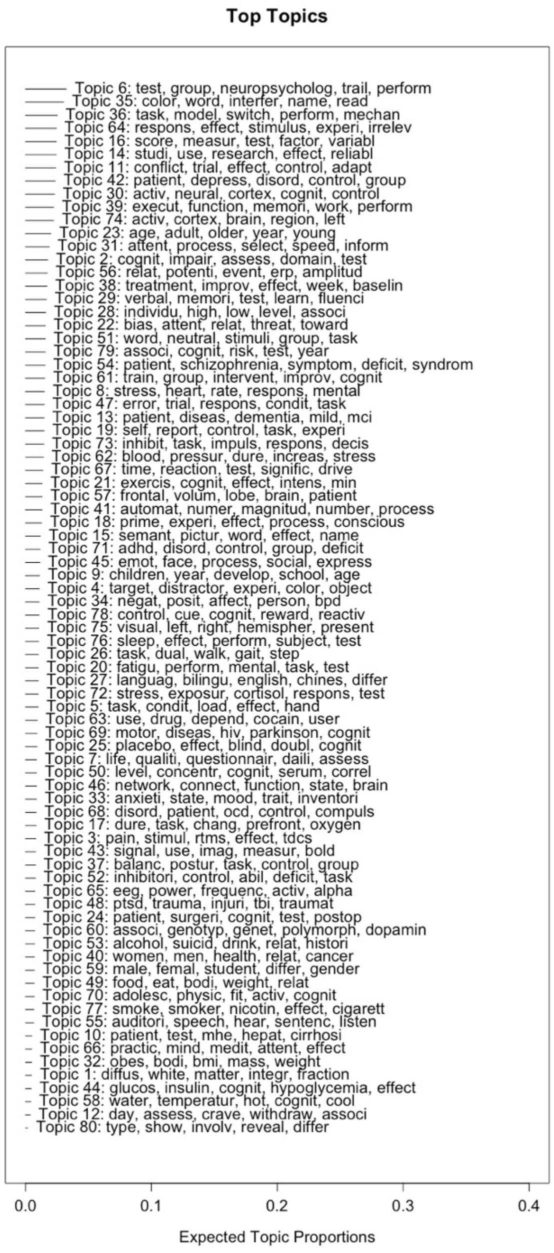

Supplementary Figure 5. Top five probable words in each topic and expected topic proportions from the STM with (**A**) Three geographic regions (North-Central America, Europe, and Asia) and (**B**) publication periods as metadata for the abstracts of the Stroop test.

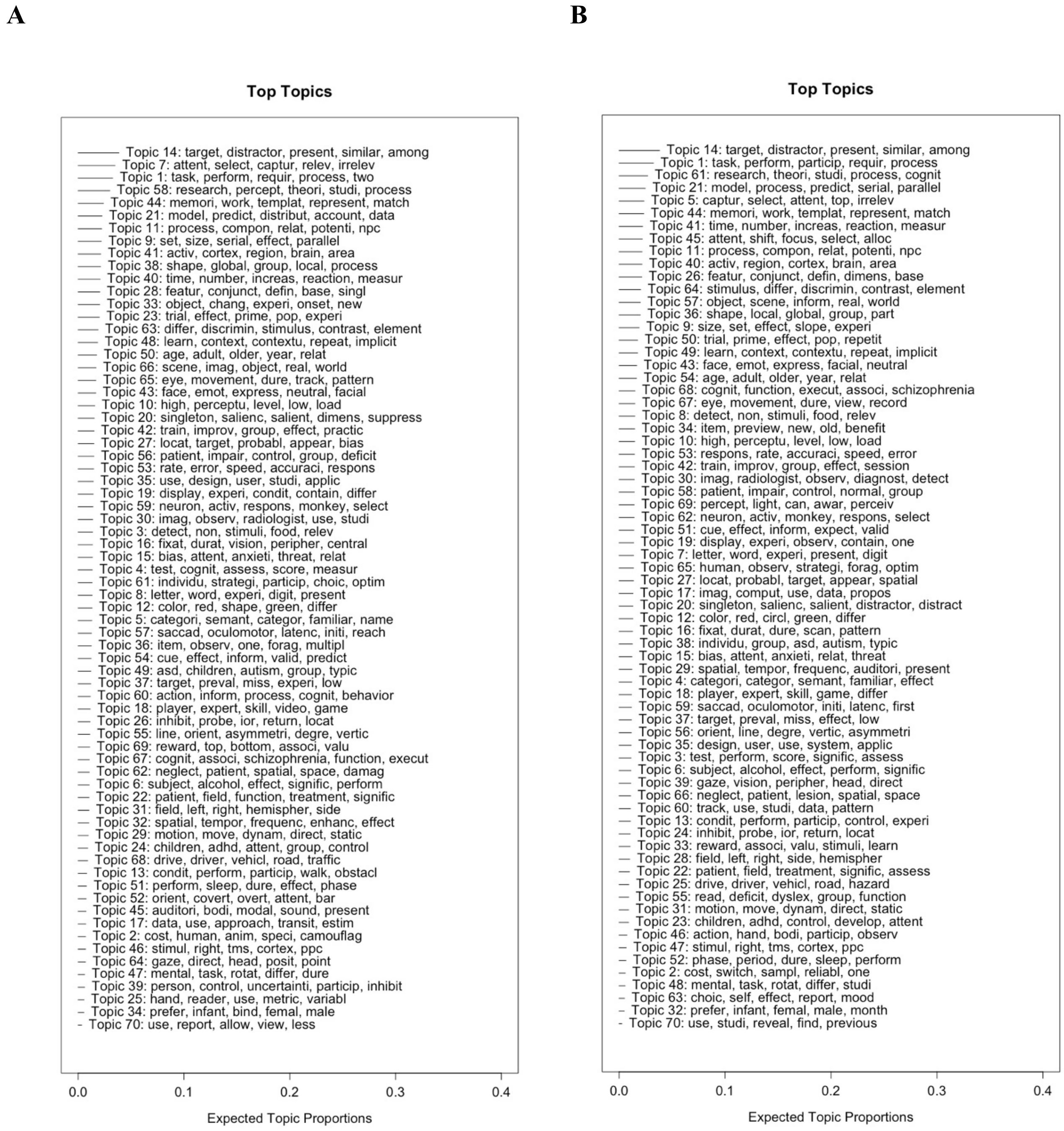

Supplementary Figure 6. Top five probable words in each topic and expected topic proportions from the STM with (**A**) Three geographic regions (North-Central America, Europe, and Asia) and (**B**) publication periods as metadata for the abstracts of visual search.

A

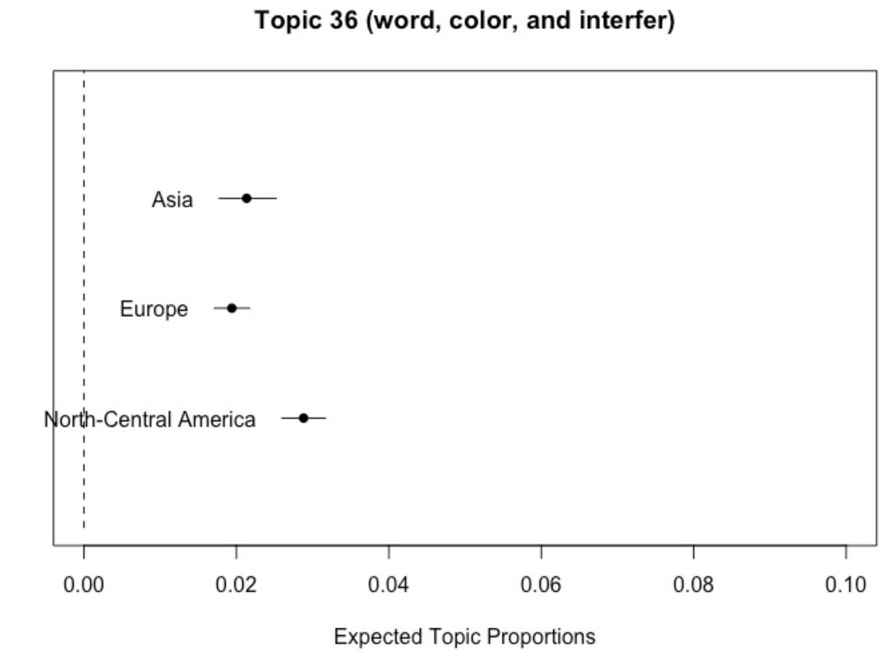
Topic 36 (word, color, and interfer)
Asia
Europe
North-Central America
0.00
0.02
0.04
0.06
0.08
0.10
Expected Topic Proportions

B

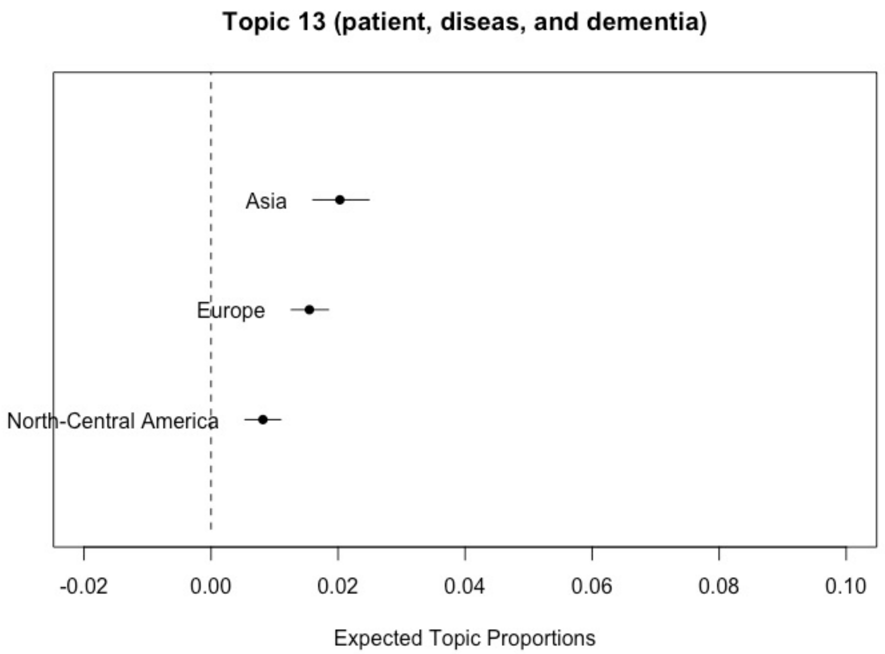
Topic 13 (patient, diseas, and dementia)
Asia
Europe
North-Central America
-0.02
0.00
0.02
0.04
0.06
0.08
0.10
Expected Topic Proportions

C

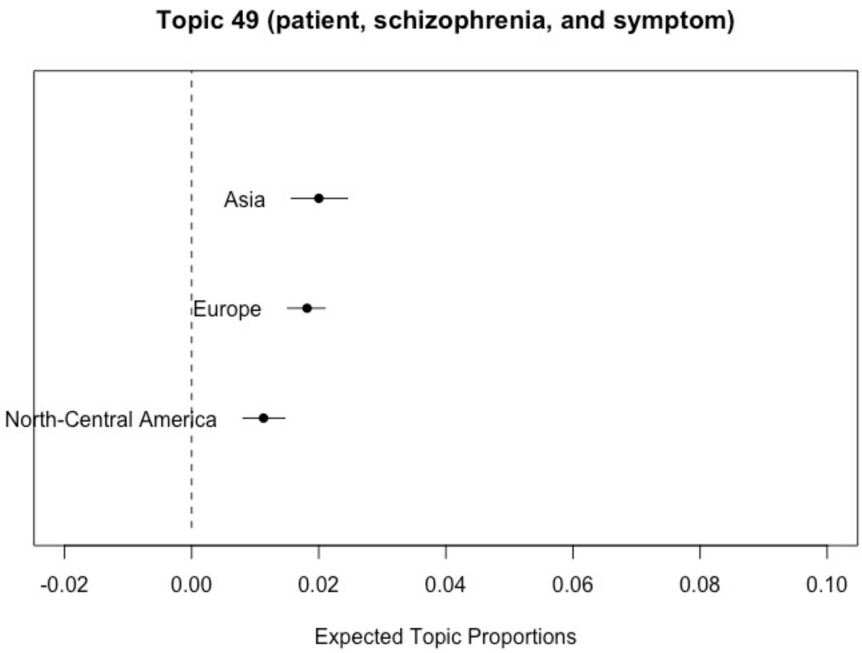

Supplementary Figure 7. Graphical displays of expected topic proportions of (**A**) Topic 36 ("word," "color," and "interfer"), (**B**) Topic 13 ("patient," "diseas," and "dementia"), and (**C**) Topic 49 ("patient," "schizophrenia," and "symptom") from the STM with 80 topics of the abstracts related to the Stroop test as a function of three geographic regions (North-Central America, Europe, and Asia).

A

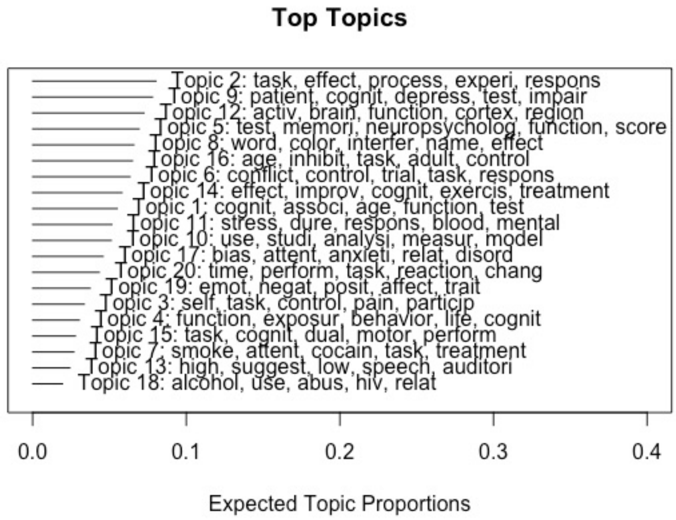
Top Topics
Topic 2: task, effect, process, experi, respons
Topic 9: patient, cognit, depress, test, impair
Topic 12: activ, brain, function, cortex, region
Topic 5: test, memori, neuropsycholog, function, score
Topic 8: word, color, interfer, name, effect
Topic 16: age, inhibit, task, adult, control
Topic 6: conflict, control, trial, task, respons
Topic 14: effect, improv, cognit, exercis, treatment
Topic 1: cognit, associ, age, function, test
Topic 11: stress, dure, respons, blood, mental
Topic 10: use, studi, analysi, measur, model
Topic 17: bias, attent, anxieti, relat, disord
Topic 20: time, perform, task, reaction, chang
Topic 19: emot, negat, posit, affect, trait
Topic 3: self, task, control, pain, particip
Topic 4: function, exposur, behavior, life, cognit
Topic 15: task, cognit, dual, motor, perform
Topic 7: smoke, attent, cocain, task, treatment
Topic 13: high, suggest, low, speech, auditori
Topic 18: alcohol, use, abus, hiv, relat
0.0
0.1
0.2
0.3
0.4
Expected Topic Proportions

B

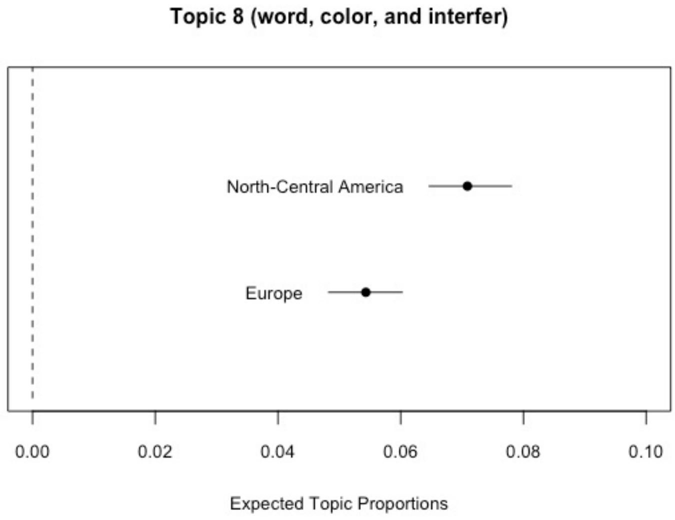
Topic 8 (word, color, and interfer)
North-Central America
Europe
0.00
0.02
0.04
0.06
0.08
0.10
Expected Topic Proportions

C

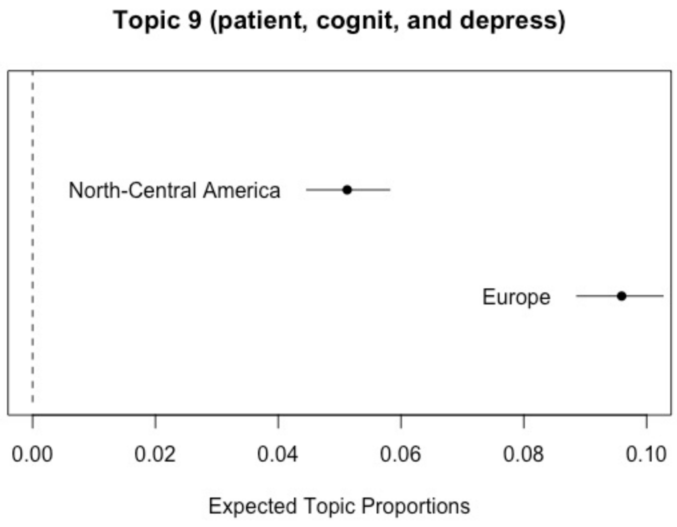

Supplementary Figure 8. Results of the STM of the manuscript abstracts related to the Stroop test with 20 topics and two geographic regions (North-Central America and Europe). (**A**) Top five probable words in each topic and expected topic proportions from the STM with two geographic regions as metadata. (**B**) Graphical display of expected topic proportions of Topic 8 (“word,” “color,” and “interfer”) as a function of two geographic regions. (**C**) Graphical display of expected topic proportions of Topic 9 (“patient,” “cognit,” and “depress”) as a function of two geographic regions.

A

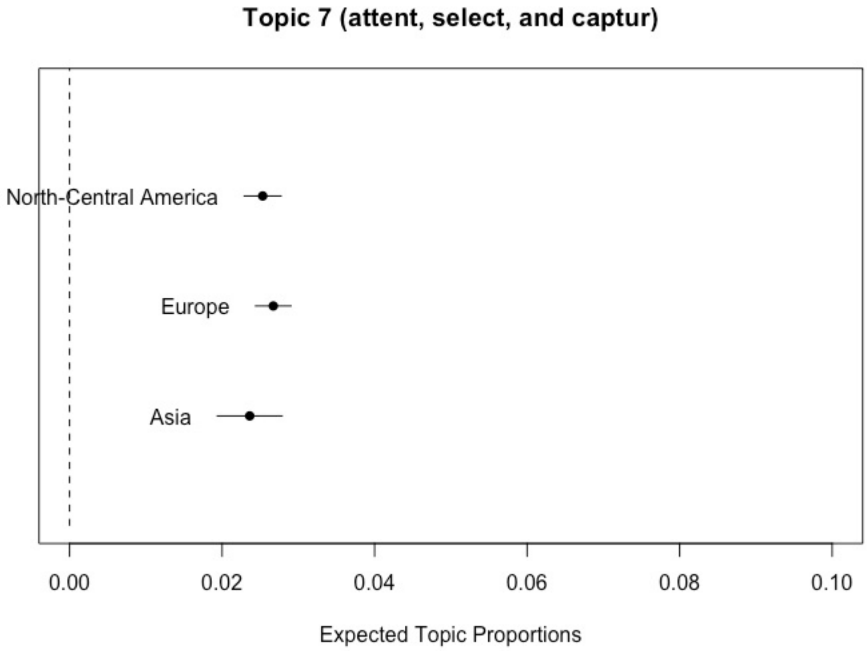
Topic 7 (attent, select, and captur)
North-Central America
Europe
Asia
0.00
0.02
0.04
0.06
0.08
0.10
Expected Topic Proportions

B

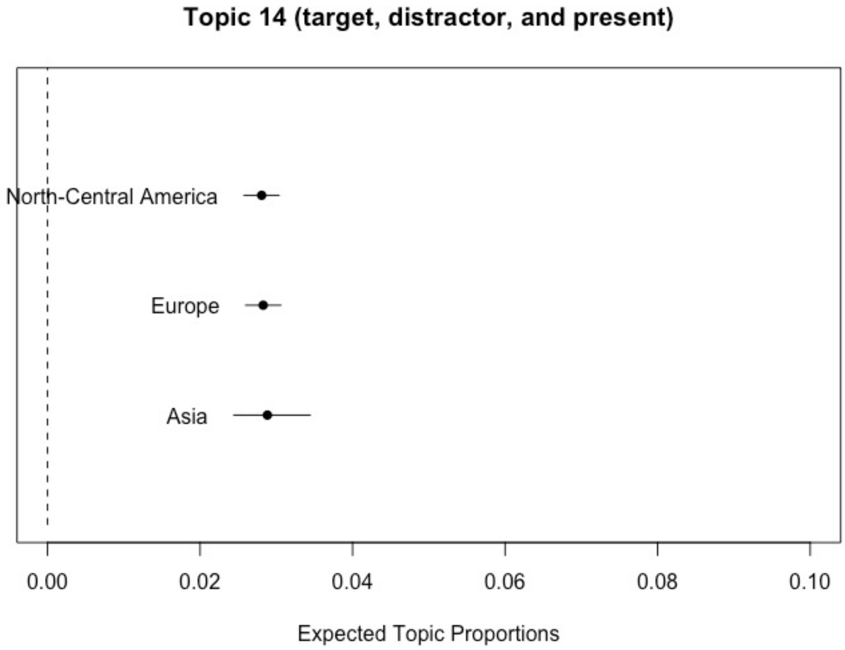
Topic 14 (target, distractor, and present)
North-Central America
Europe
Asia
0.00
0.02
0.04
0.06
0.08
0.10
Expected Topic Proportions

C

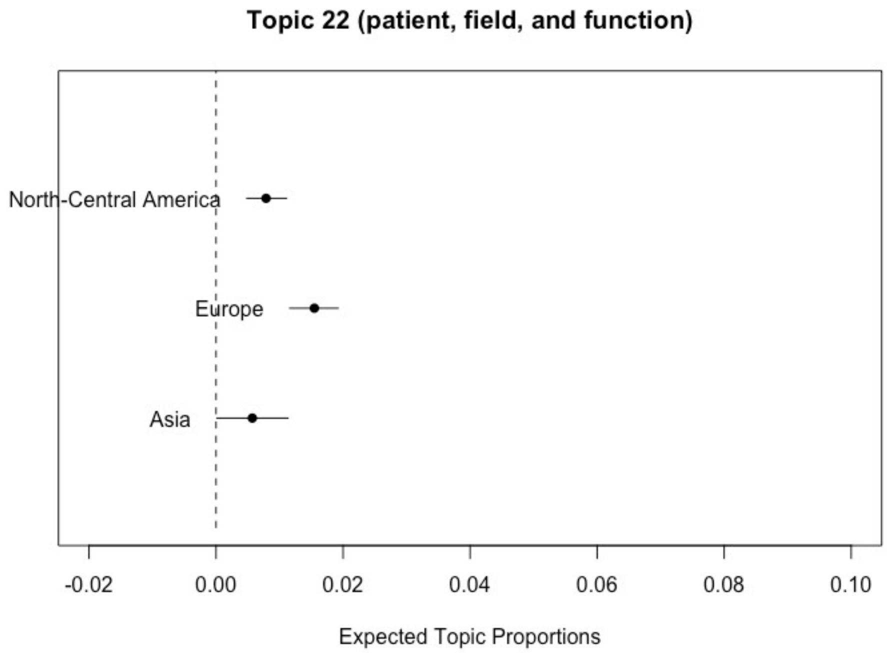

D

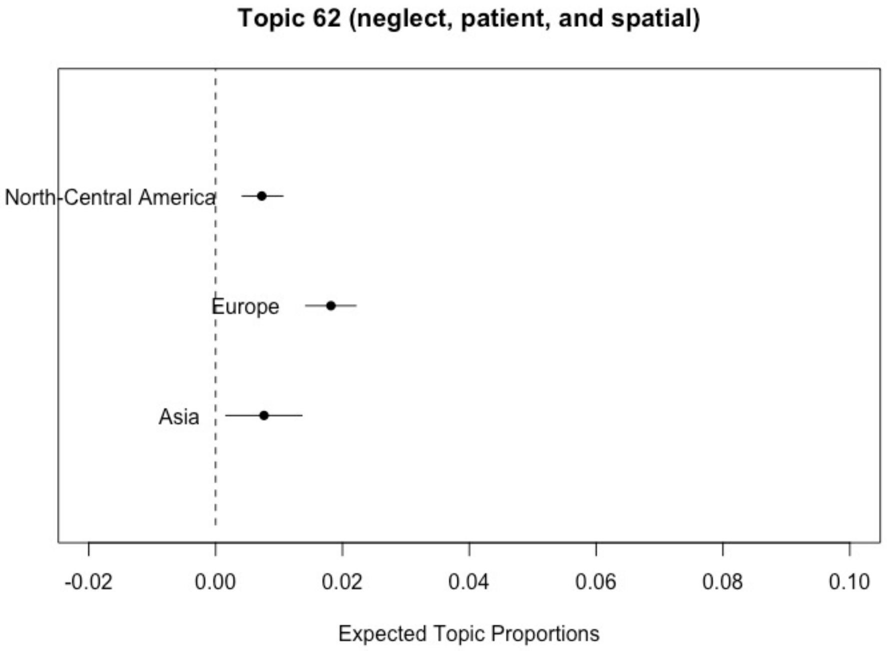

Supplementary Figure 9. Graphical displays of expected topic proportions of (**A**) Topic 7 (“attent,” “select,” and “captur”), (**B**) Topic 14 (“target,” “distractor,” and “present”), (**C**) Topic 22 (“patient,” “field,” and “function”), and (**D**) Topic 62 (“neglect,” “patient,” and “spatial”) from the STM with 70 topics of the manuscript abstracts related to visual search as a function of three geographic regions (North-Central America, Europe, and Asia).

A

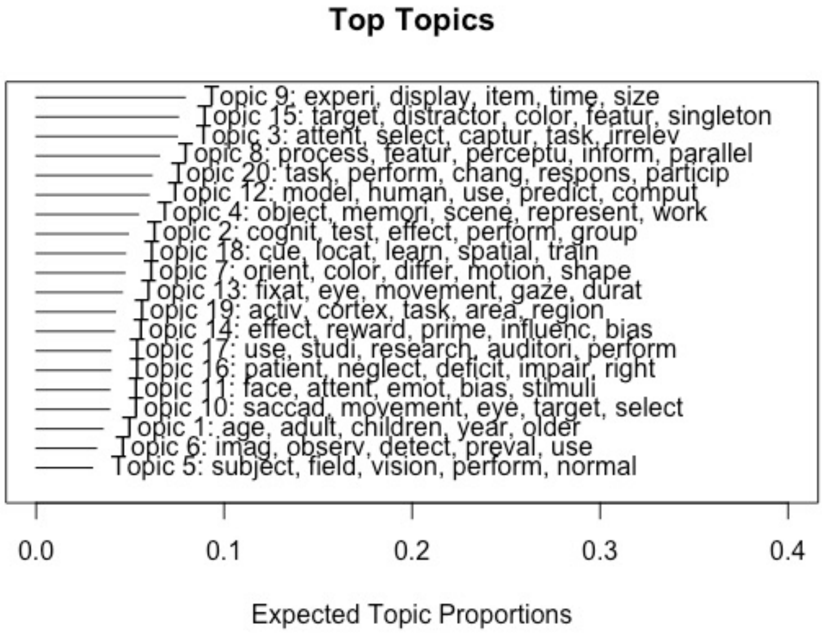
Top Topics
Topic 9: experi, display, item, time, size
Topic 15: target, distractor, color, featur, singleton
Topic 3: attent, select, captur, task, irrelev
Topic 8: process, featur, perceptu, inform, parallel
Topic 20: task, perform, chang, respons, particip
Topic 12: model, human, use, predict, comput
Topic 4: object, memori, scene, represent, work
Topic 2: cognit, test, effect, perform, group
Topic 18: cue, locat, learn, spatial, train
Topic 7: orient, color, differ, motion, shape
Topic 13: fixat, eye, movement, gaze, durat
Topic 19: activ, cortex, task, area, region
Topic 14: effect, reward, prime, influenc, bias
Topic 17: use, studi, research, auditori, perform
Topic 16: patient, neglect, deficit, impair, right
Topic 11: face, attent, emot, bias, stimuli
Topic 10: saccad, movement, eye, target, select
Topic 1: age, adult, children, year, older
Topic 6: imag, observ, detect, preval, use
Topic 5: subject, field, vision, perform, normal
0.0
0.1
0.2
0.3
0.4
Expected Topic Proportions

B

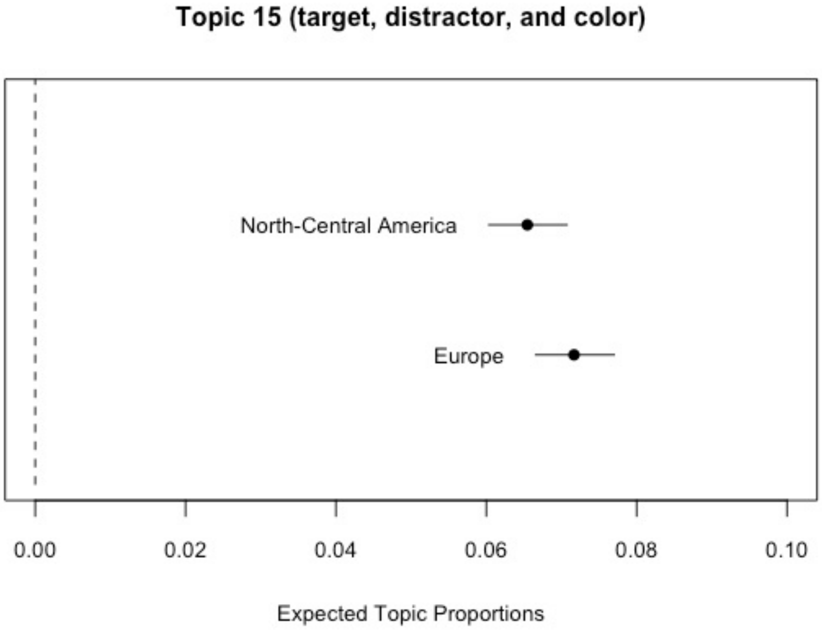
Topic 15 (target, distractor, and color)
North-Central America
Europe
0.00
0.02
0.04
0.06
0.08
0.10
Expected Topic Proportions

C

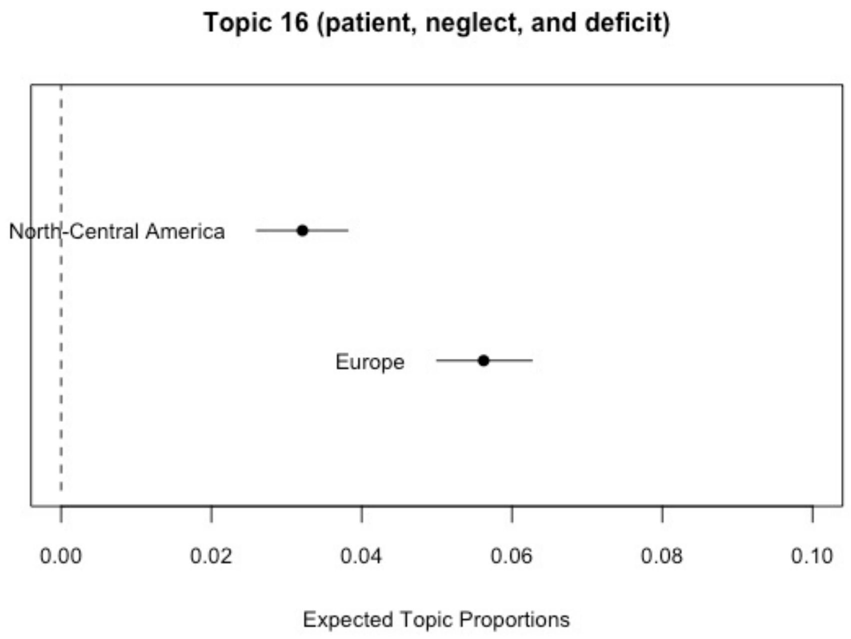

Supplementary Figure 10. Results of the STM of the abstracts related to visual search with 20 topics and two geographic regions (North-Central America and Europe). (**A**) Top five probable words in each topic and expected topic proportions from the STM with two geographic regions as meta-data. (**B**) Graphical display of expected topic proportions of Topic 15 (“target,” “distractor,” and “color”) as a function of two geographic regions. (**C**) Graphical display of expected topic proportions of Topic 16 (“patient,” “neglect,” and “deficit”) as a function of two geographic regions.

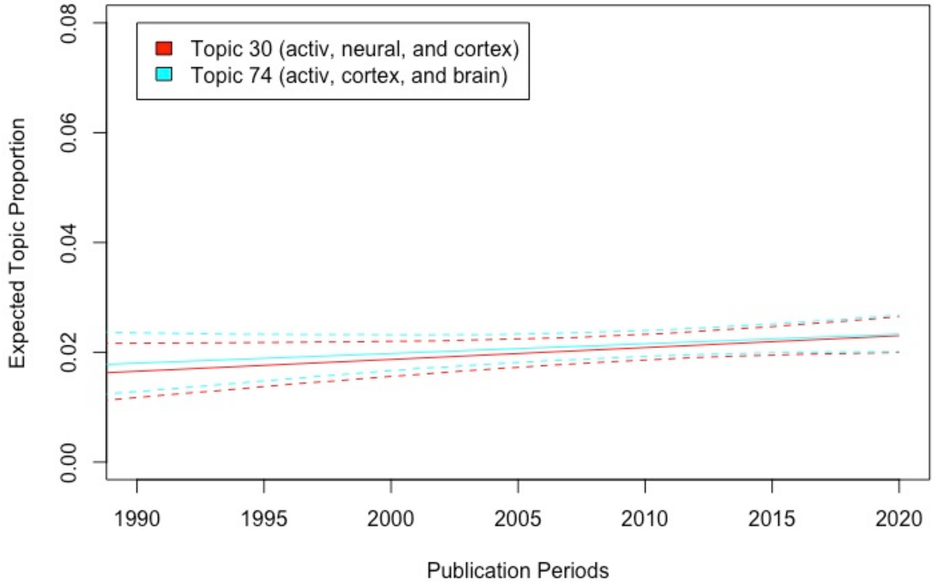

Supplementary Figure 11. Graphical display of expected topic proportions of Topic 30 ("activ," "neural," and "cortex") and Topic 74 ("activ," "cortex," and "brain") as a function of publication periods from the STM with 80 topics of the manuscript abstracts related to the Stroop test.

A

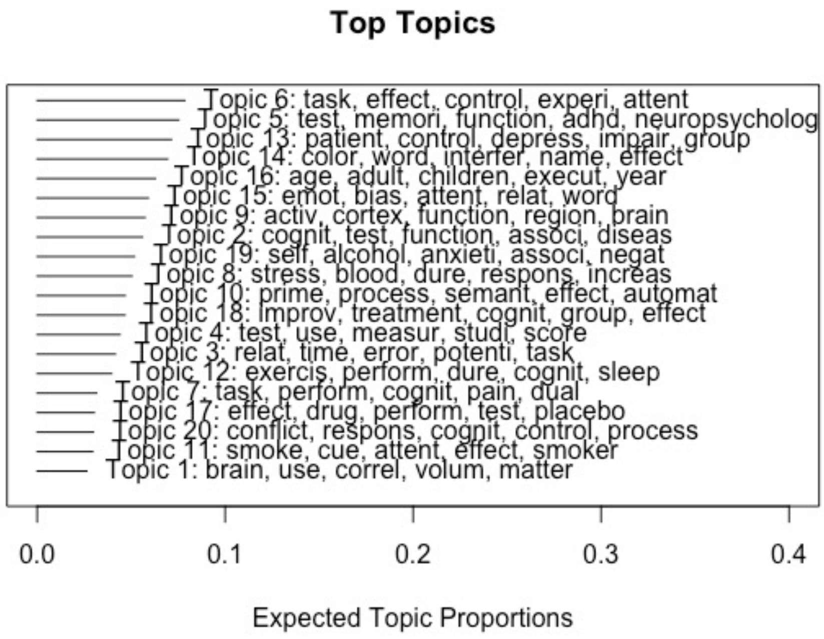

B

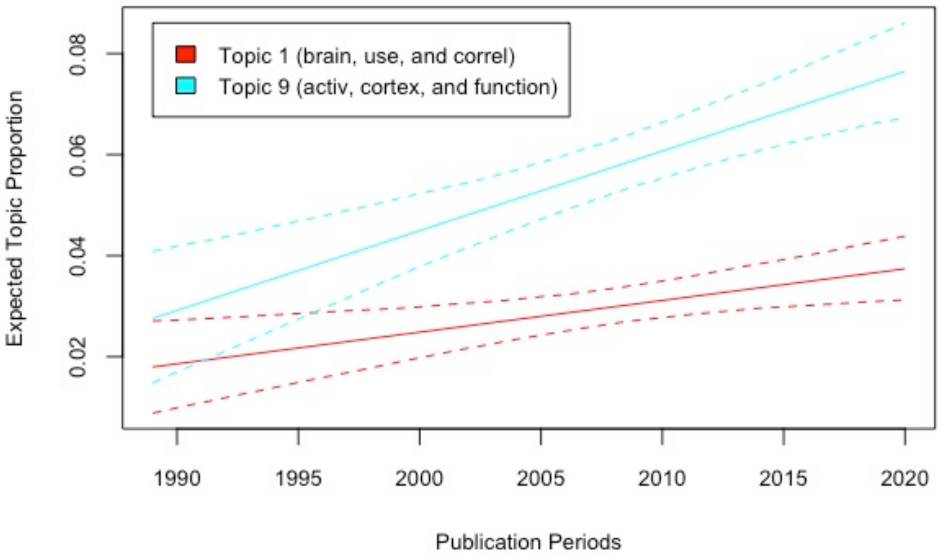

Supplementary Figure 12. Results of the STM of the manuscript abstracts related to the Stroop test with 20 topics and down-sampling of the number of abstracts in each publication period to 218. (**A**) Top five probable words in each topic and expected topic proportions from the STM. (**B**) Graphical display of expected topic proportions of Topic 1 ("brain," "use," and "correl") and Topic 9 ("activ," "cortex," and "function") as a function of publication periods.

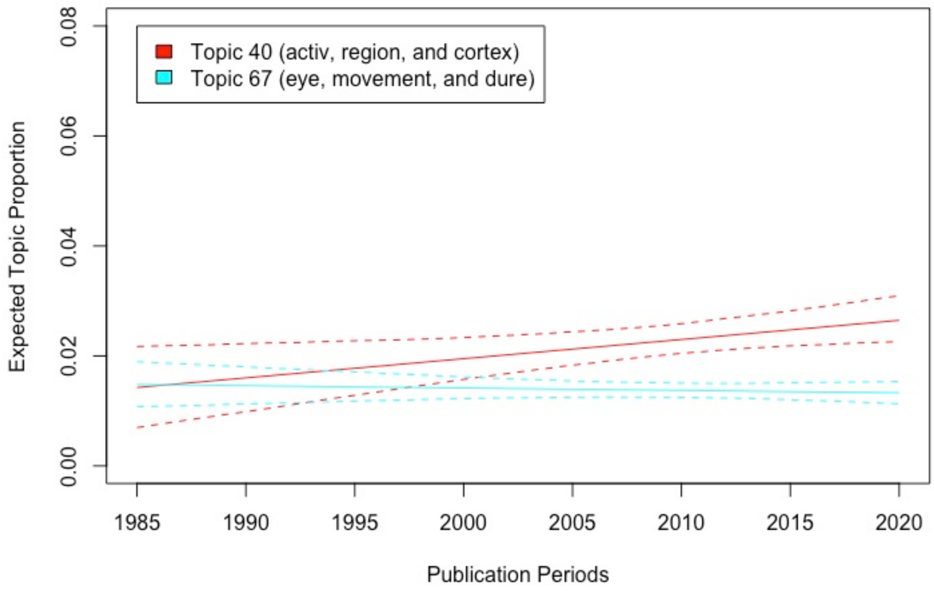

Supplementary Figure 13. Graphical displays of expected topic proportions of Topic 40 ("activ," "region," and "cortex") and Topic 67 ("eye," "movement," and "dure") as a function of publication periods from the STM with 70 topics of the manuscript abstracts related to visual search as a function of publication periods.

A

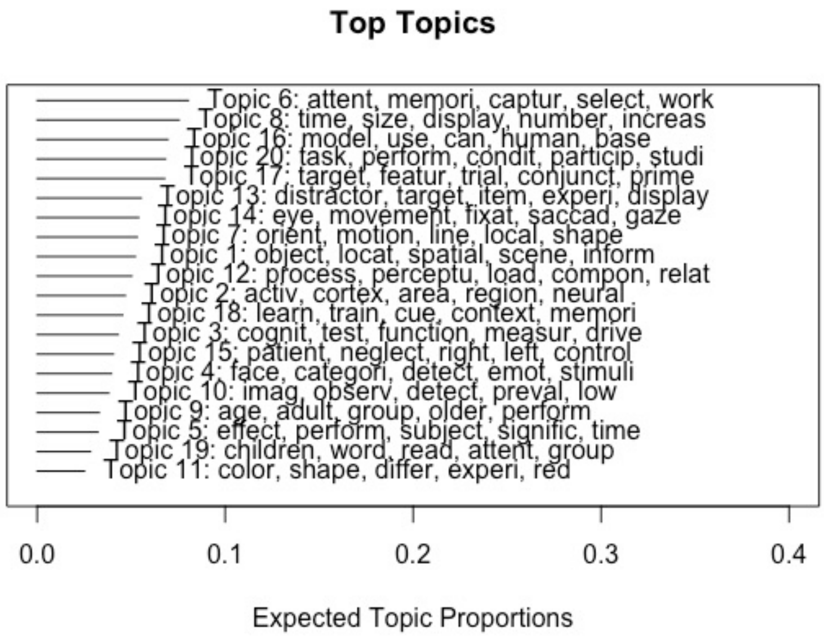

B

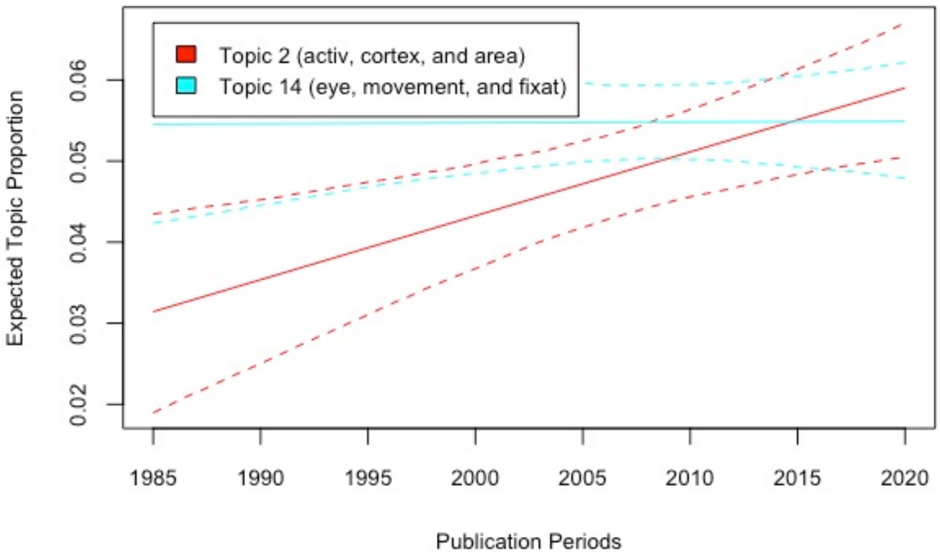

Supplementary Figure 14. Results of the STM of the manuscript abstracts related to visual search with 20 topics and down-sampling of the number of abstracts in each publication period to 203. (**A**) Top five probable words in each topic and expected topic proportions from the STM. (**B**) Graphical display of expected topic proportions of Topic 2 (“activ,” “cortex,” and “area”) and Topic 14 (“eye,” “movement,” and “fixat”) as a function of publication periods.

A

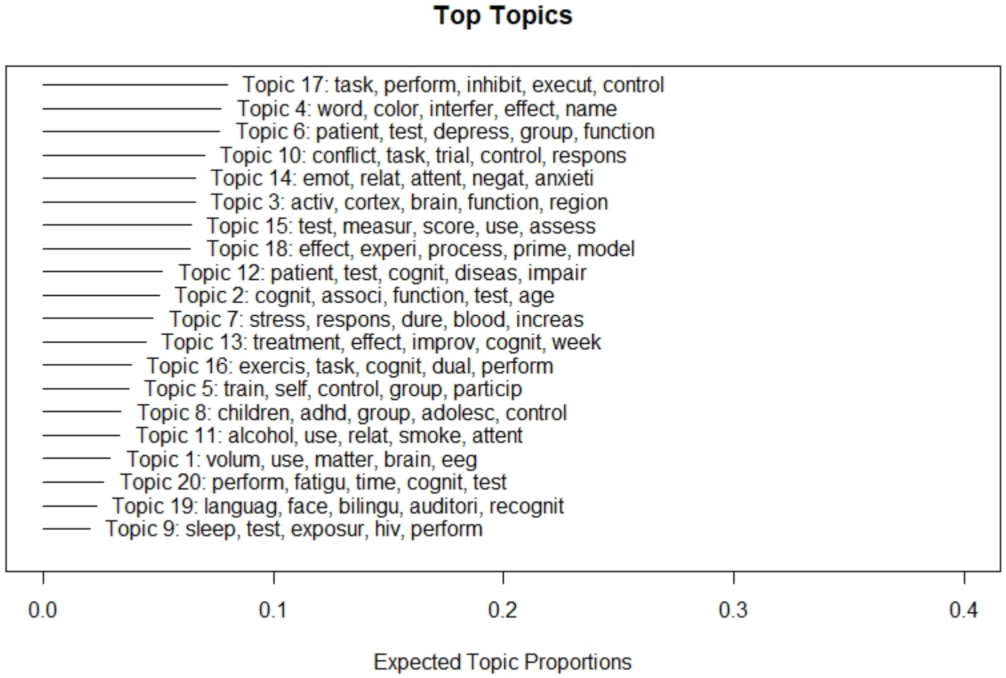
Top Topics
Topic 17: task, perform, inhibit, execut, control
Topic 4: word, color, interfer, effect, name
Topic 6: patient, test, depress, group, function
Topic 10: conflict, task, trial, control, respons
Topic 14: emot, relat, attent, negat, anxieti
Topic 3: activ, cortex, brain, function, region
Topic 15: test, measur, score, use, assess
Topic 18: effect, experi, process, prime, model
Topic 12: patient, test, cognit, diseas, impair
Topic 2: cognit, associ, function, test, age
Topic 7: stress, respons, dure, blood, increas
Topic 13: treatment, effect, improv, cognit, week
Topic 16: exercis, task, cognit, dual, perform
Topic 5: train, self, control, group, particip
Topic 8: children, adhd, group, adolesc, control
Topic 11: alcohol, use, relat, smoke, attent
Topic 1: volum, use, matter, brain, eeg
Topic 20: perform, fatigu, time, cognit, test
Topic 19: languag, face, bilingu, auditori, recognit
Topic 9: sleep, test, exposur, hiv, perform
0.0
0.1
0.2
0.3
0.4
Expected Topic Proportions

B

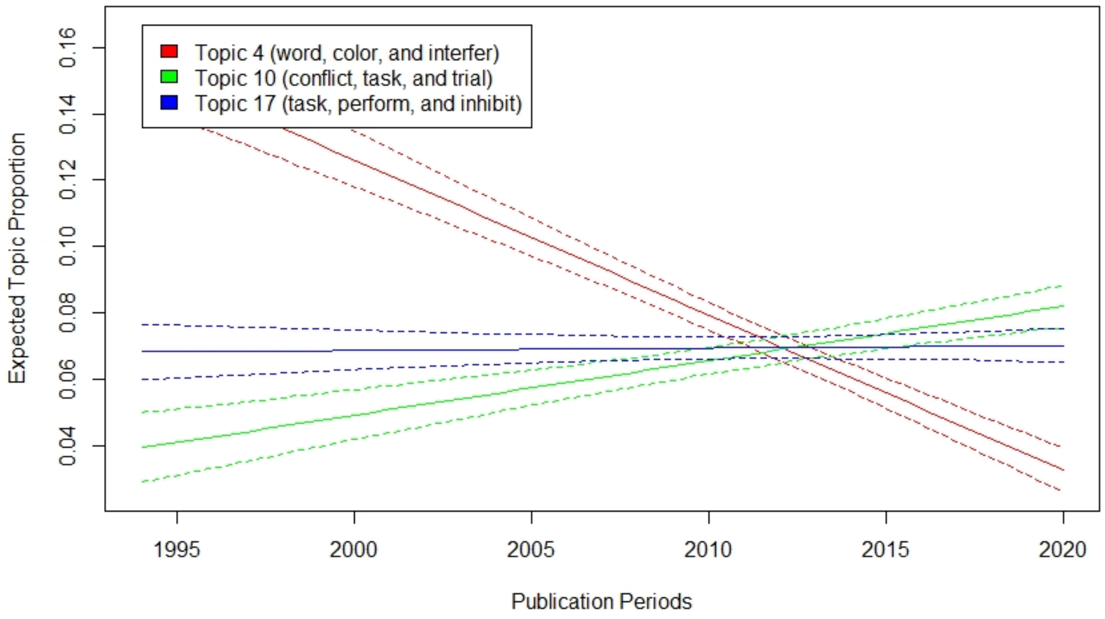
Topic 4 (word, color, and interfer)
Topic 10 (conflict, task, and trial)
Topic 17 (task, perform, and inhibit)
Expected Topic Proportion
0.04
0.06
0.08
0.10
0.12
0.14
0.16
1995
2000
2005
2010
2015
2020
Publication Periods

C

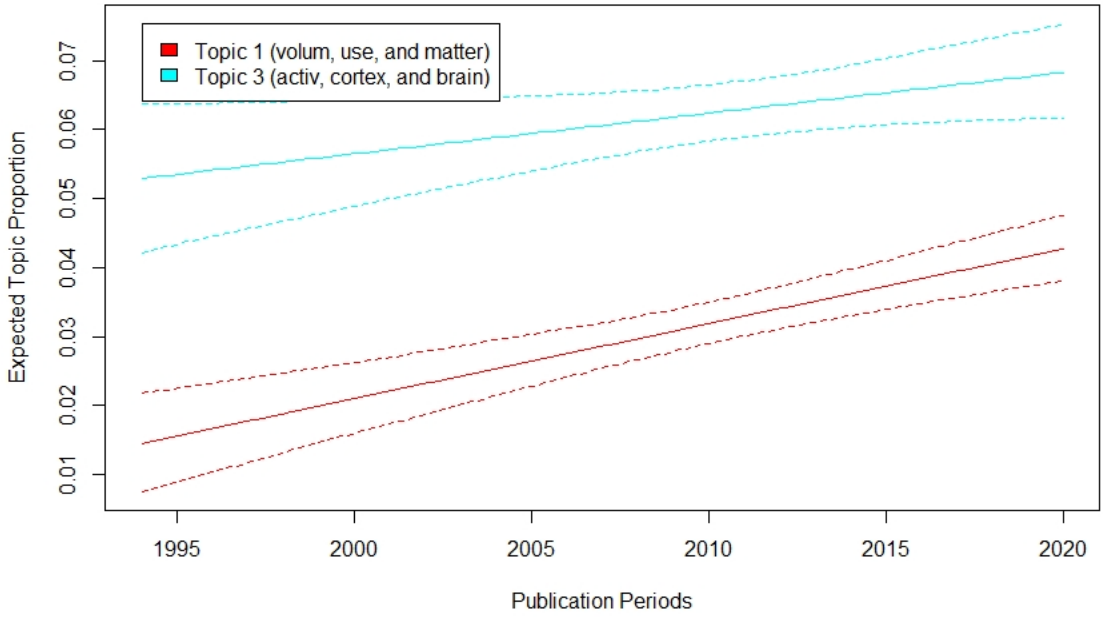

Supplementary Figure 15. Results of the STM of the manuscript abstracts related to the Stroop test excluding those published before 1990. We observed similar patterns of trends to the main results. (**A**) Top five probable words in each topic and expected topic proportions from the STM. (**B**) Graphical display of expected topic proportions of Topic 4 ("word," "color," and "interfer"), Topic 10 ("conflict," "task," and "trial"), and Topic 17 ("task," "perform," and "inhibit") as a function of publication periods. (**C**) Graphical display of expected topic proportions of Topic 1 ("volum," "use," and "matter") and Topic 3 ("activ," "cortex," and "brain") as a function of publication periods.

A

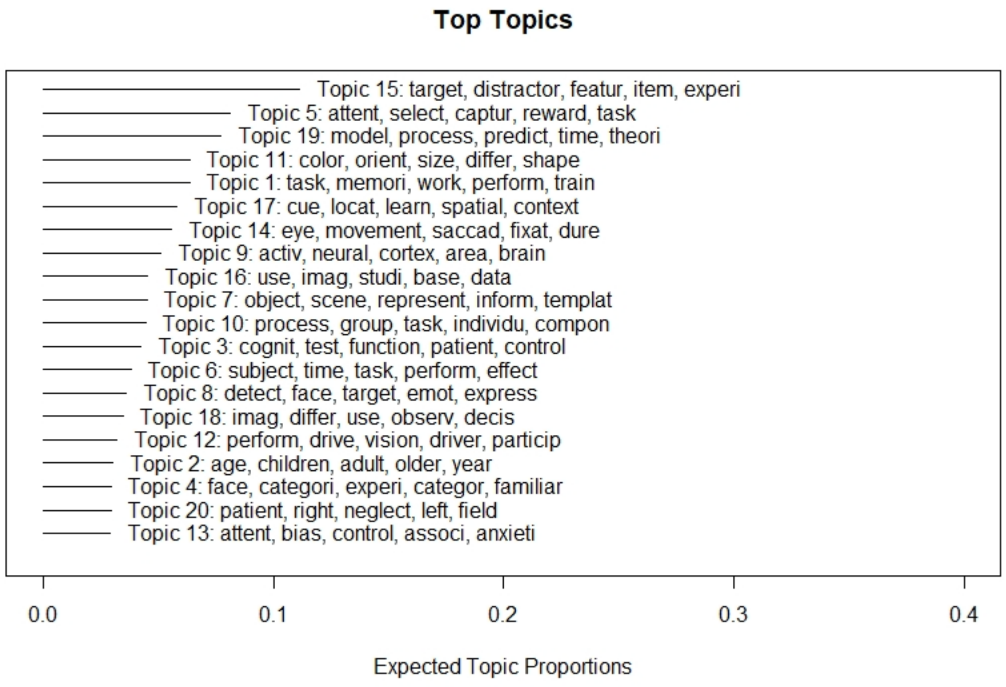
Top Topics
Topic 15: target, distractor, featur, item, experi
Topic 5: attent, select, captur, reward, task
Topic 19: model, process, predict, time, theori
Topic 11: color, orient, size, differ, shape
Topic 1: task, memori, work, perform, train
Topic 17: cue, locat, learn, spatial, context
Topic 14: eye, movement, saccad, fixat, dure
Topic 9: activ, neural, cortex, area, brain
Topic 16: use, imag, studi, base, data
Topic 7: object, scene, represent, inform, templat
Topic 10: process, group, task, individu, compon
Topic 3: cognit, test, function, patient, control
Topic 6: subject, time, task, perform, effect
Topic 8: detect, face, target, emot, express
Topic 18: imag, differ, use, observ, decis
Topic 12: perform, drive, vision, driver, particip
Topic 2: age, children, adult, older, year
Topic 4: face, categori, experi, categor, familiar
Topic 20: patient, right, neglect, left, field
Topic 13: attent, bias, control, associ, anxieti
0.0
0.1
0.2
0.3
0.4
Expected Topic Proportions

B

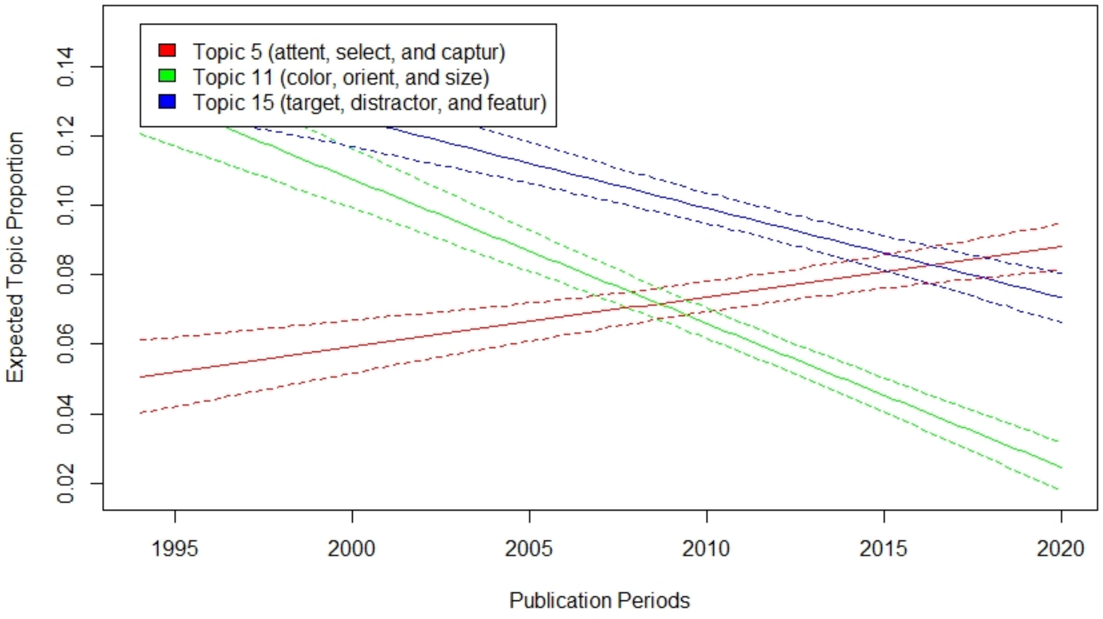
Topic 5 (attent, select, and captur)
Topic 11 (color, orient, and size)
Topic 15 (target, distractor, and featur)
Expected Topic Proportion
0.02
0.04
0.06
0.08
0.10
0.12
0.14
1995
2000
2005
2010
2015
2020
Publication Periods

C

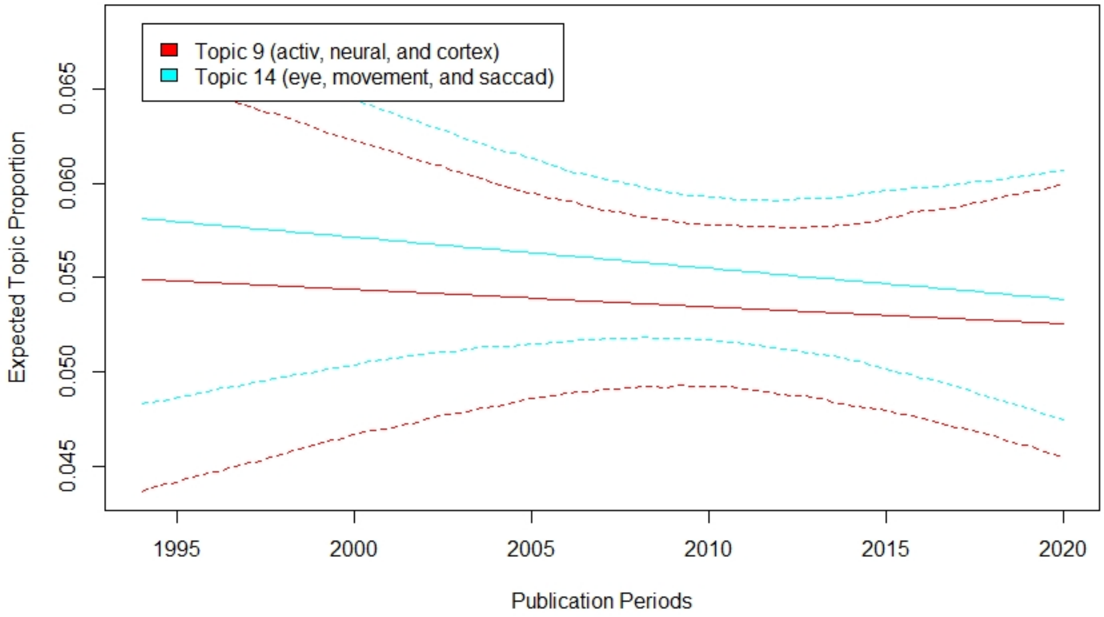

Supplementary Figure 16. Results of the STM of the manuscript abstracts related to visual search excluding those published before 1990. Contrary to the main results, we did not observe significant trends of publication periods in the topic related to neuroscience research. This result suggests that there are increasing trends of neuroscience studies from pre-1990 to post-1990. (**A**) Top five probable words in each topic and expected topic proportions from the STM. (**B**) Graphical display of expected topic proportions of Topic 5 ("attent," "select," and "captur"), Topic 11 ("color," "orient," and "size"), and Topic 15 ("target," "distractor," and "featur") as a function of publication periods. (**C**) Graphical display of expected topic proportions of Topic 9 ("activ," "neural," and "cortex") and Topic 14 ("eye," "movement," and "saccad") as a function of publication periods.

A

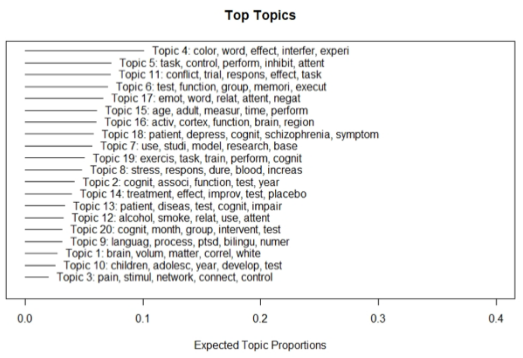
Top Topics
Topic 4: color, word, effect, interfer, experi
Topic 5: task, control, perform, inhibit, attent
Topic 11: conflict, trial, respons, effect, task
Topic 6: test, function, group, memori, execut
Topic 17: emot, word, relat, attent, negat
Topic 15: age, adult, measur, time, perform
Topic 16: activ, cortex, function, brain, region
Topic 18: patient, depress, cognit, schizophrenia, symptom
Topic 7: use, studi, model, research, base
Topic 19: exercis, task, train, perform, cognit
Topic 8: stress, respons, dure, blood, increas
Topic 2: cognit, associ, function, test, year
Topic 14: treatment, effect, improv, test, placebo
Topic 13: patient, diseas, test, cognit, impair
Topic 12: alcohol, smoke, relat, use, attent
Topic 20: cognit, month, group, intervent, test
Topic 9: languag, process, ptsd, bilingu, numer
Topic 1: brain, volum, matter, correl, white
Topic 10: children, adolesc, year, develop, test
Topic 3: pain, stimul, network, connect, control
0.0
0.1
0.2
0.3
0.4
Expected Topic Proportions

B

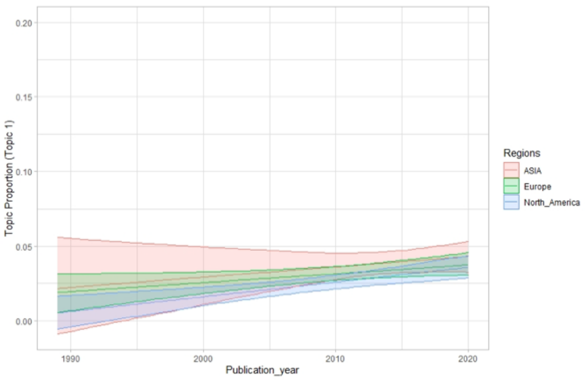
Topic Proportion (Topic 1)
Publication_year
Regions
ASIA
Europe
North_America

C

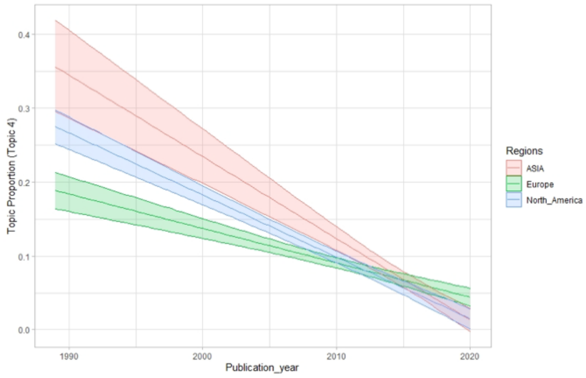
Topic Proportion (Topic 4)
Publication_year
Regions
ASIA
Europe
North_America

D

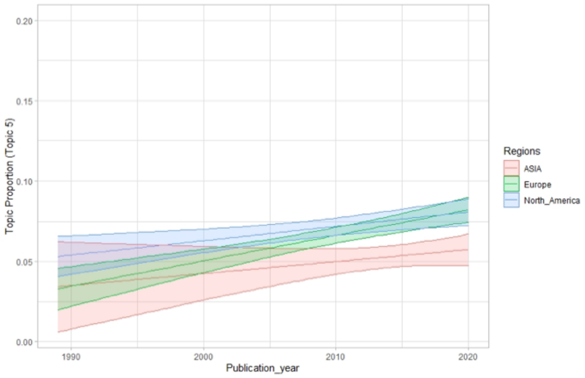
Topic Proportion (Topic 5)
Publication_year
Regions
ASIA
Europe
North_America

E

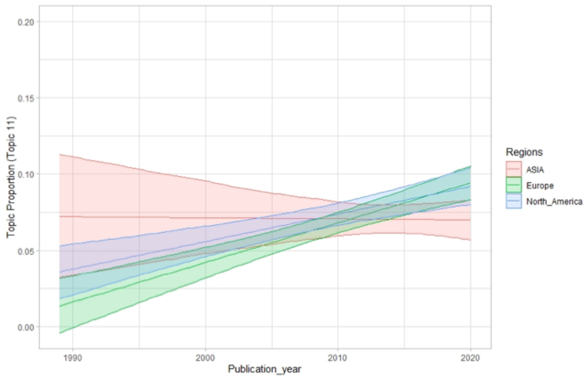
Topic Proportion (Topic 11)
Publication_year
Regions
ASIA
Europe
North_America

F

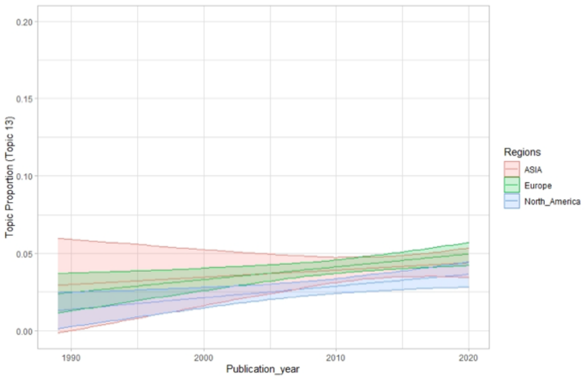
Topic Proportion (Topic 13)
Publication_year
Regions
ASIA
Europe
North_America

G

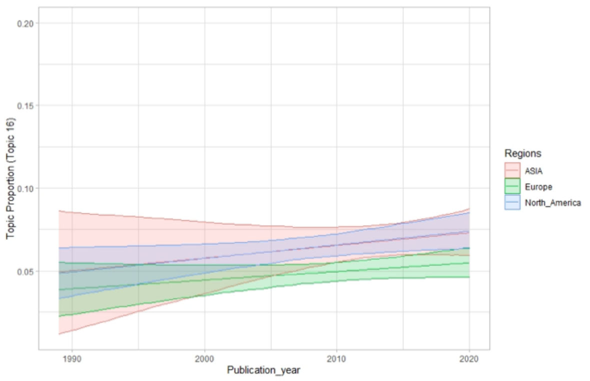

H

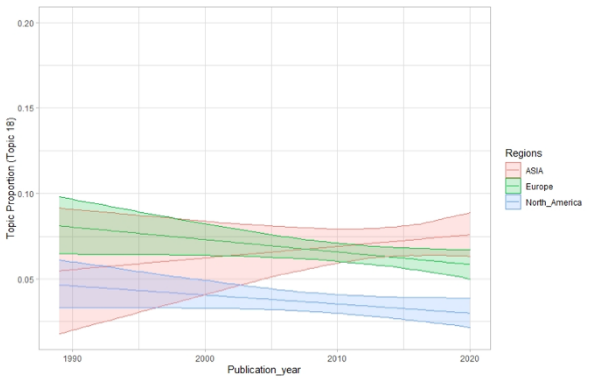

Supplementary Figure 17. Results of the STM of the manuscript abstracts related to the Stroop test with the interaction term between the geographic regions and publication periods. (**A**) Top five probable words in each topic and expected topic proportions from the STM. (**B**) Graphical display of expected topic proportions of Topic 1 ("brain," "volum," and "matter") as a function of geographic regions and publication periods. (**C**) Graphical display of expected topic proportions of Topic 4 ("color," "word," and "effect") as a function of geographic regions and publication periods. (**D**) Graphical display of expected topic proportions of Topic 5 ("task," "control," and "perform") as a function of geographic regions and publication periods. (**E**) Graphical display of expected topic proportions of Topic 11 ("conflict," "trial," and "respons") as a function of geographic regions and publication periods. (**F**) Graphical display of expected topic proportions of Topic 13 ("patient," "diseas," and "test") as a function of geographic regions and publication periods. (**G**) Graphical display of expected topic proportions of Topic 16 ("activ," "cortex," and "function") as a function of geographic regions and publication periods. (**H**) Graphical display of expected topic proportions of Topic 18 ("patient," "depress," and "cognit") as a function of geographic regions and publication periods.

A

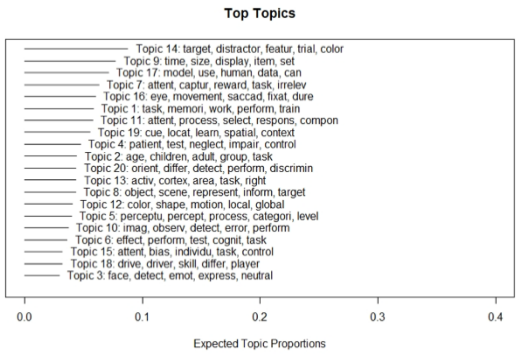
Top Topics
Topic 14: target, distractor, featur, trial, color
Topic 9: time, size, display, item, set
Topic 17: model, use, human, data, can
Topic 7: attent, captur, reward, task, irrelev
Topic 16: eye, movement, saccad, fixat, dure
Topic 1: task, memori, work, perform, train
Topic 11: attent, process, select, respons, compon
Topic 19: cue, locat, learn, spatial, context
Topic 4: patient, test, neglect, impair, control
Topic 2: age, children, adult, group, task
Topic 20: orient, differ, detect, perform, discrimin
Topic 13: activ, cortex, area, task, right
Topic 8: object, scene, represent, inform, target
Topic 12: color, shape, motion, local, global
Topic 5: perceptu, percept, process, categori, level
Topic 10: imag, observ, detect, error, perform
Topic 6: effect, perform, test, cognit, task
Topic 15: attent, bias, individu, task, control
Topic 18: drive, driver, skill, differ, player
Topic 3: face, detect, emot, express, neutral
0.0
0.1
0.2
0.3
0.4
Expected Topic Proportions

B

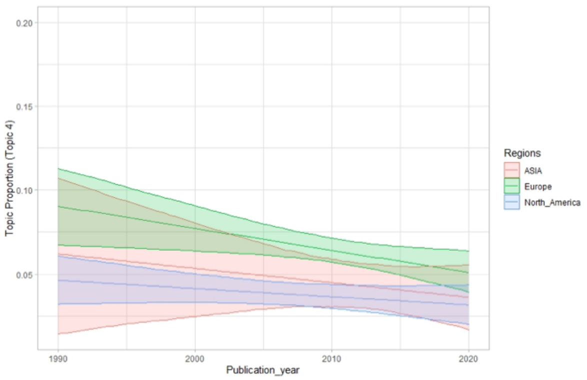
Topic Proportion (Topic 4)
Publication_year
Regions
ASIA
Europe
North_America

C

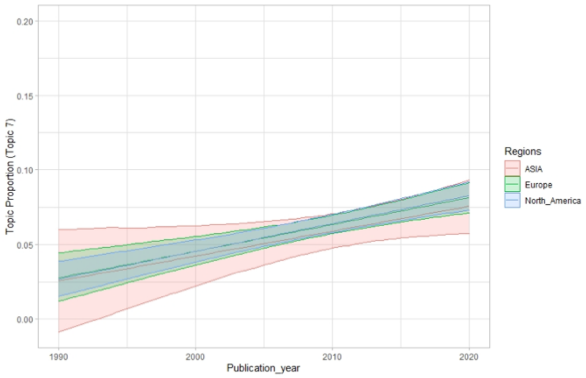
Topic Proportion (Topic 7)
Publication_year
Regions
ASIA
Europe
North_America

D

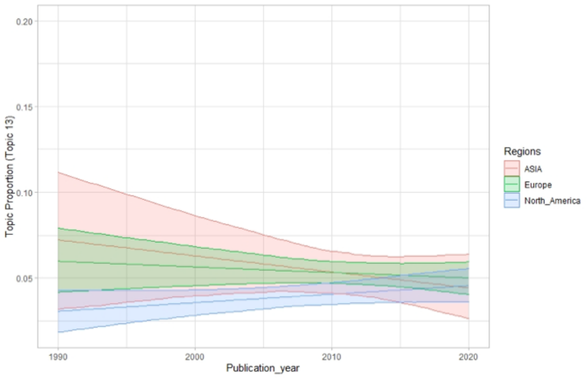
Topic Proportion (Topic 13)
Publication_year
Regions
ASIA
Europe
North_America

E

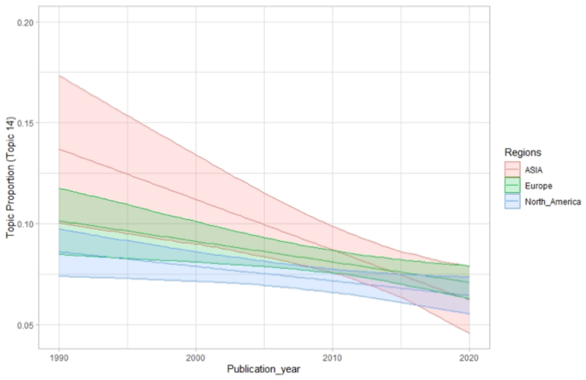
Topic Proportion (Topic 14)
Publication_year
Regions
ASIA
Europe
North_America

F

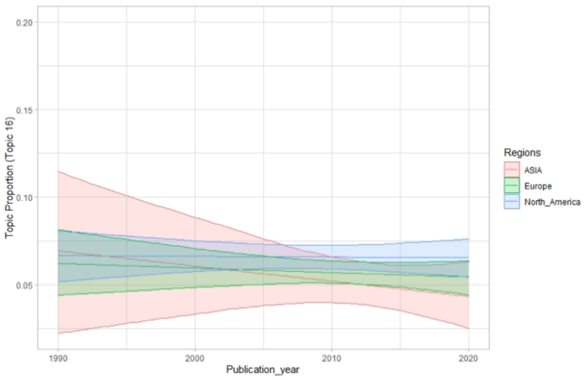
Topic Proportion (Topic 16)
Publication_year
Regions
ASIA
Europe
North_America

G

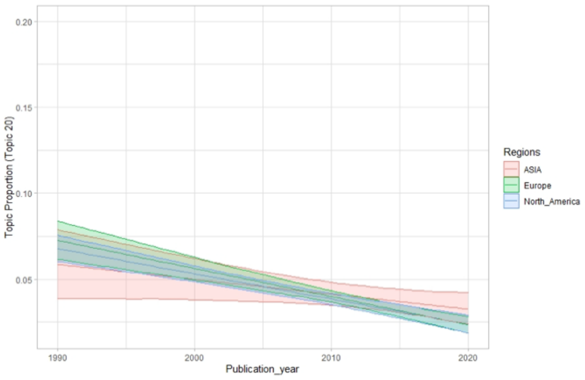

Supplementary Figure 18. Results of the STM of the manuscript abstracts related to visual search with the interaction term between the geographic regions and publication periods. (**A**) Top five probable words in each topic and expected topic proportions from the STM. (**B**) Graphical display of expected topic proportions of Topic 4 ("patient," "test," and "neglect") as a function of geographic regions and publication periods. (**C**) Graphical display of expected topic proportions of Topic 7 ("attent," "captur," and "reward") as a function of geographic regions and publication periods. (**D**) Graphical display of expected topic proportions of Topic 13 ("activ," "cortex," and "area") as a function of geographic regions and publication periods. (**E**) Graphical display of expected topic proportions of Topic 14 ("target," "distractor," and "featur") as a function of geographic regions and publication periods. (**F**) Graphical display of expected topic proportions of Topic 16 ("eye," "movement," and "saccad") as a function of geographic regions and publication periods. (**G**) Graphical display of expected topic proportions of Topic 20 ("orient," "differ," and "detect") as a function of geographic regions and publication periods.

A

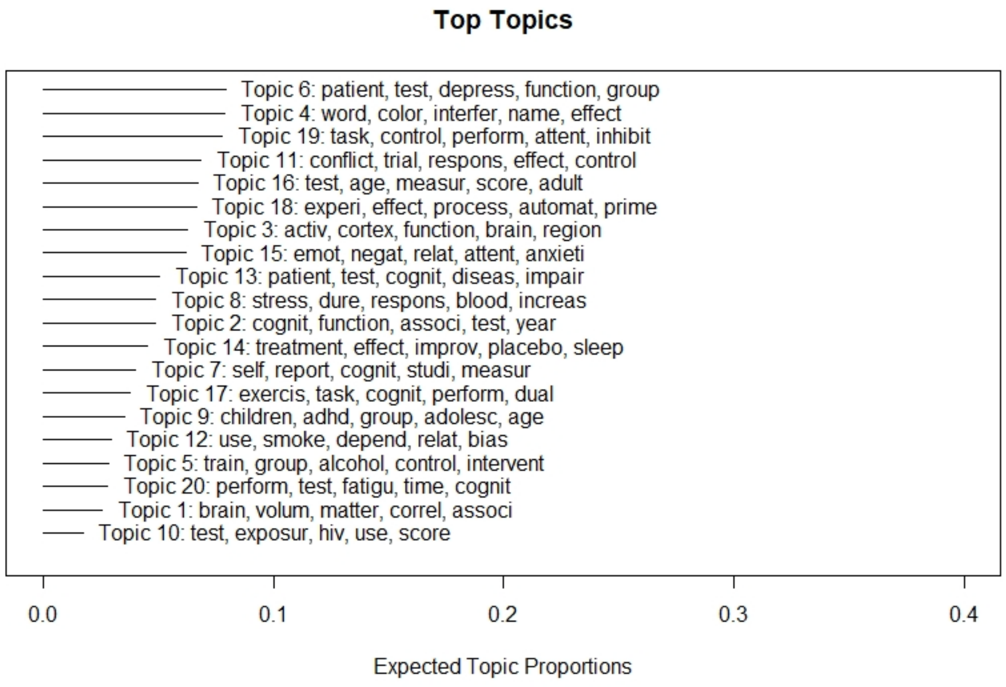
Top Topics
Topic 6: patient, test, depress, function, group
Topic 4: word, color, interfer, name, effect
Topic 19: task, control, perform, attent, inhibit
Topic 11: conflict, trial, respons, effect, control
Topic 16: test, age, measur, score, adult
Topic 18: experi, effect, process, automat, prime
Topic 3: activ, cortex, function, brain, region
Topic 15: emot, negat, relat, attent, anxieti
Topic 13: patient, test, cognit, diseas, impair
Topic 8: stress, dure, respons, blood, increas
Topic 2: cognit, function, associ, test, year
Topic 14: treatment, effect, improv, placebo, sleep
Topic 7: self, report, cognit, studi, measur
Topic 17: exercis, task, cognit, perform, dual
Topic 9: children, adhd, group, adolesc, age
Topic 12: use, smoke, depend, relat, bias
Topic 5: train, group, alcohol, control, intervent
Topic 20: perform, test, fatigu, time, cognit
Topic 1: brain, volum, matter, correl, associ
Topic 10: test, exposur, hiv, use, score
0.0
0.1
0.2
0.3
0.4
Expected Topic Proportions

B

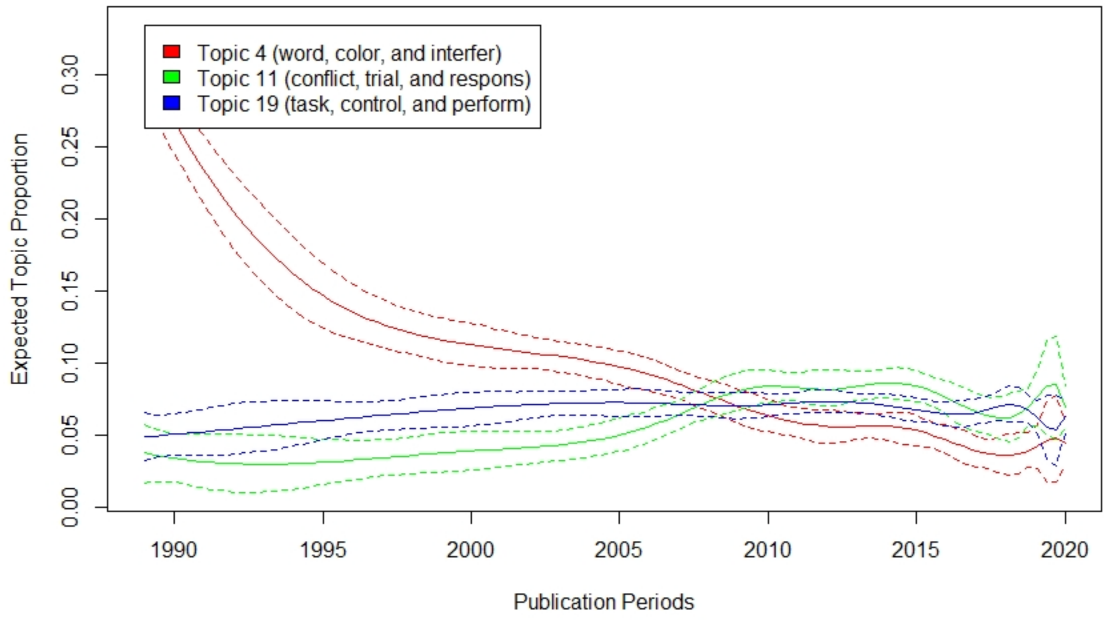
Topic 4 (word, color, and interfer)
Topic 11 (conflict, trial, and respons)
Topic 19 (task, control, and perform)
Expected Topic Proportion
0.00
0.05
0.10
0.15
0.20
0.25
0.30
1990
1995
2000
2005
2010
2015
2020
Publication Periods

C

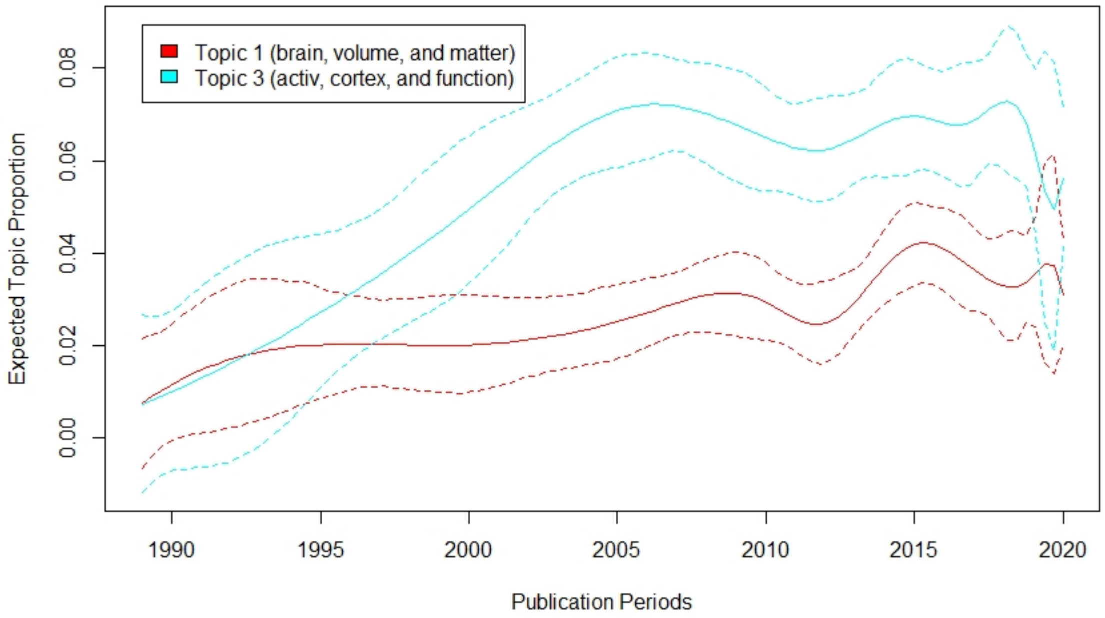

Supplementary Figure 19. Non-linear results of the STM of the manuscript abstracts related to the Stroop test. We observed similar patterns of trends to the linear results. (**A**) Top five probable words in each topic and expected topic proportions from the STM. (**B**) Graphical display of expected topic proportions of Topic 4 (“word,” “color,” and “interfer”), Topic 11 (“conflict,” “trial,” and “respons”), and Topic 19 (“task,” “control,” and “perform”) as a function of publication periods. (**C**) Graphical display of expected topic proportions of Topic 1 (“brain,” “volum,” and “matter”) and Topic 3 (“activ,” “cortex,” and “function”) as a function of publication periods.

A

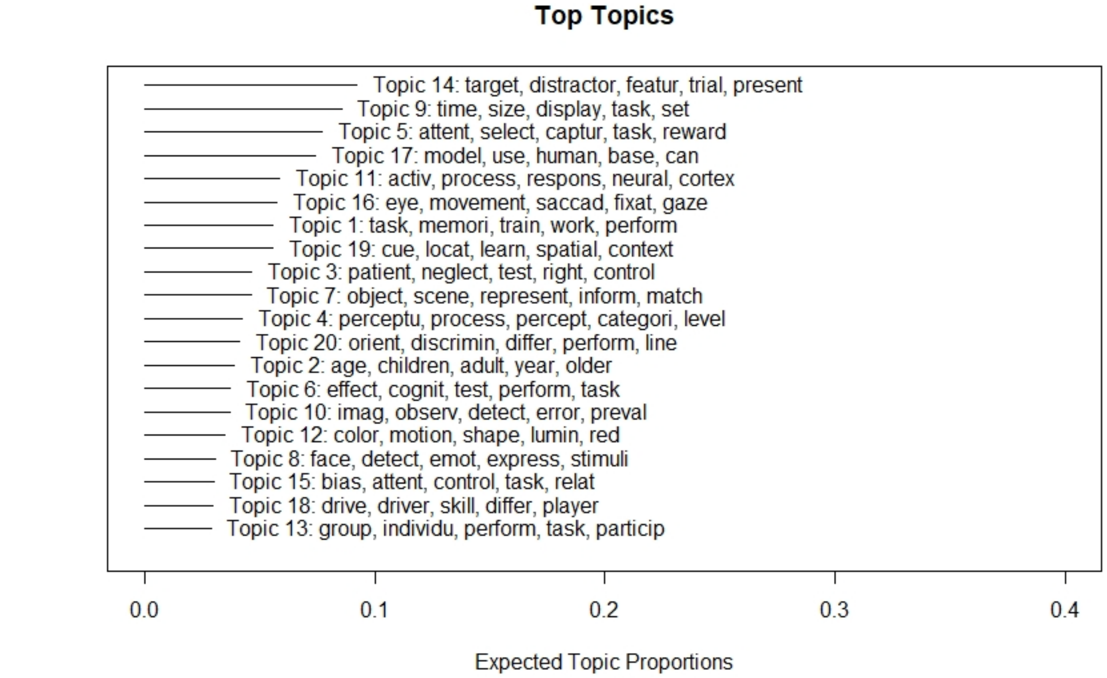
Top Topics
Topic 14: target, distractor, featur, trial, present
Topic 9: time, size, display, task, set
Topic 5: attent, select, captur, task, reward
Topic 17: model, use, human, base, can
Topic 11: activ, process, respons, neural, cortex
Topic 16: eye, movement, saccad, fixat, gaze
Topic 1: task, memori, train, work, perform
Topic 19: cue, locat, learn, spatial, context
Topic 3: patient, neglect, test, right, control
Topic 7: object, scene, represent, inform, match
Topic 4: perceptu, process, percept, categori, level
Topic 20: orient, discrimin, differ, perform, line
Topic 2: age, children, adult, year, older
Topic 6: effect, cognit, test, perform, task
Topic 10: imag, observ, detect, error, preval
Topic 12: color, motion, shape, lumin, red
Topic 8: face, detect, emot, express, stimuli
Topic 15: bias, attent, control, task, relat
Topic 18: drive, driver, skill, differ, player
Topic 13: group, individu, perform, task, particip
0.0
0.1
0.2
0.3
0.4
Expected Topic Proportions

B

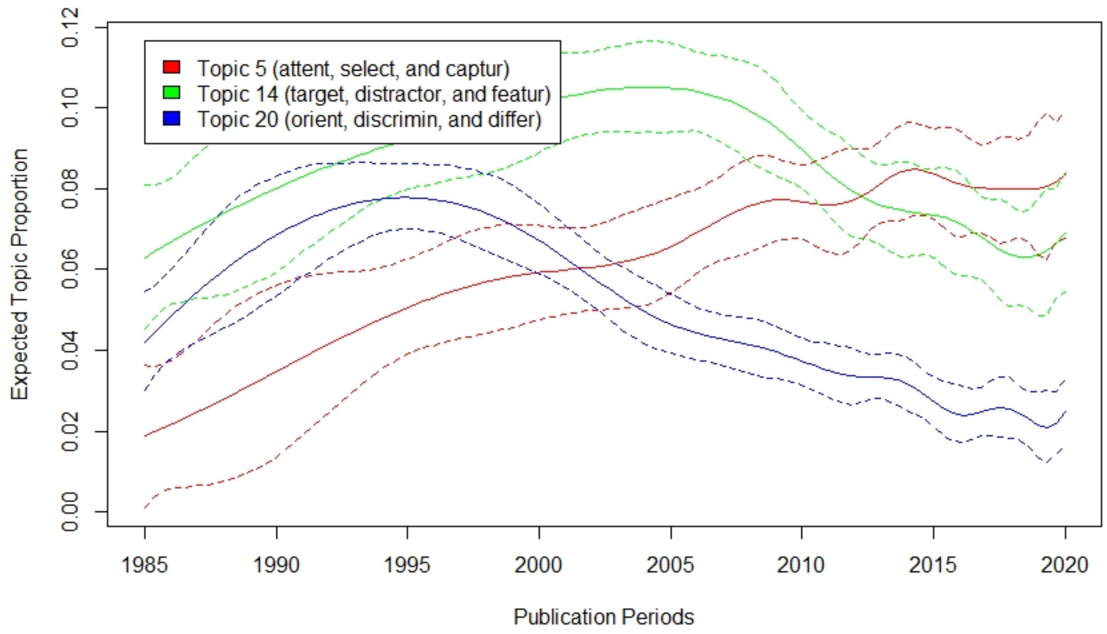
Topic 5 (attent, select, and captur)
Topic 14 (target, distractor, and featur)
Topic 20 (orient, discrimin, and differ)
Expected Topic Proportion
0.00
0.02
0.04
0.06
0.08
0.10
0.12
1985
1990
1995
2000
2005
2010
2015
2020
Publication Periods

C

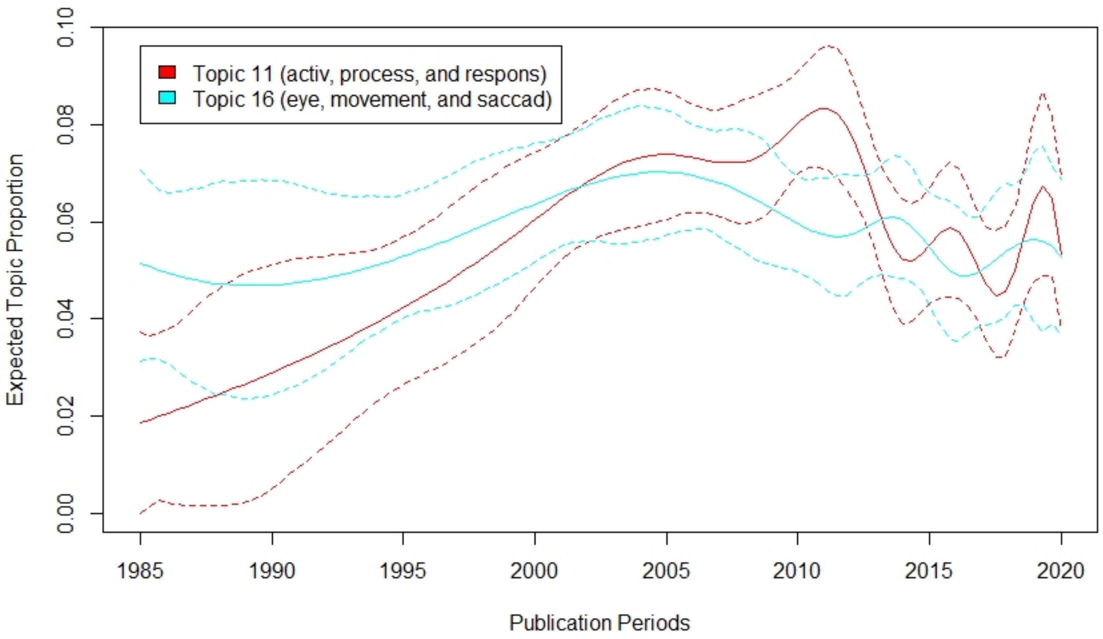

Supplementary Figure 20. Non-linear results of the STM of the manuscript abstracts related to visual search. We observed similar patterns of trends to the linear results. (**A**) Top five probable words in each topic and expected topic proportions from the STM. (**B**) Graphical display of expected topic proportions of Topic 5 ("attent," "select," and "captur"), Topic 14 ("target," "distractor," and "featur"), and Topic 20 ("orient," "discrimin," and "differ") as a function of publication periods. (**C**) Graphical display of expected topic proportions of Topic 11 ("activ," "process," and "respons") and Topic 16 ("eye," "movement," and "saccad") as a function of publication periods.

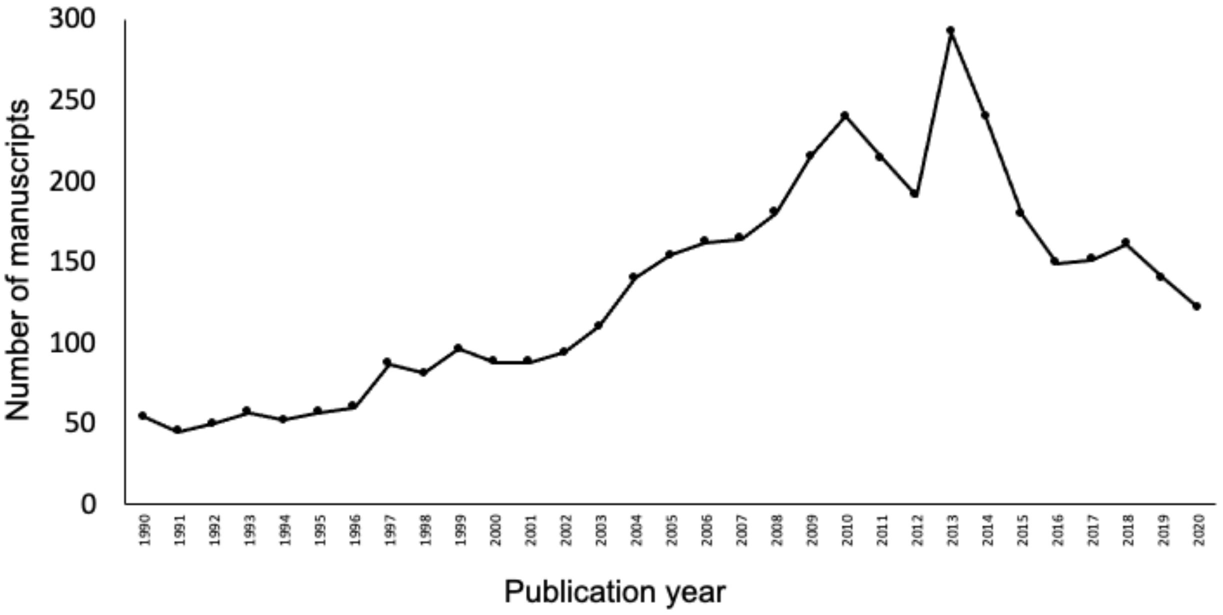

Supplementary Figure 21. Number of manuscripts published in Psychological Science from 1990 to 2020.
